# Berberine Alleviates Lipopolysaccharide‐Induced Impairments in Neuroplasticity and Spatial Memory by Modulating Microglial Polarization via MAPK Signaling Inhibition

**DOI:** 10.1155/np/6795481

**Published:** 2026-02-05

**Authors:** Lirong Jiang, Ruiyi Liao, Jiaxin Wang, Yuan Yang, Jiaojiao Sun, Li Liu, Yiwei Wang, Wei Dong, Yang Yu

**Affiliations:** ^1^ Department of Histology and Embryology, School of Basic Medical Sciences, Southwest Medical University, Luzhou, 646000, Sichuan, China, swmu.edu.cn; ^2^ Key Laboratory of Medical Electrophysiology, Ministry of Education and Medical Electrophysiological Key Laboratory of Sichuan Province, Institute of Cardiovascular Research, Southwest Medical University, Luzhou, 646000, Sichuan, China, swmu.edu.cn; ^3^ Department of Medical Imaging Technology, Nanyang Medical College, Nanyang, 473004, Henan, China; ^4^ State Key Laboratory of Biotherapy and Cancer Center, West China Hospital, Sichuan University, Chengdu, 610041, China, scu.edu.cn

**Keywords:** berberine, cognitive impairment, MAPK signaling, microglia, neuroinflammation

## Abstract

Neuroinflammation‐induced cognitive impairment is characterized by a continued decline in memory, executive functioning, and information‐processing abilities. Although berberine (BBR) exhibits anti‐inflammatory and neuroprotective properties, its ability to mitigate cognitive deficits by regulating microglial‐mediated neuroinflammation remains incompletely understood. To investigate the potential of BBR in mitigating microglial‐mediated neuroinflammation and its detrimental effects on neuroplasticity and spatial memory, a mouse model was established through intrahippocampal microinjection of lipopolysaccharide (LPS). The results showed that BBR pretreatment significantly improved cognitive performance, suppressed microglial activation, reduced hippocampal neuronal damage, and increased the density of functional dendritic spines. Mechanistic analysis revealed that BBR treatment inhibited the phosphorylation of key proteins in the MAPK signaling pathway within microglia. These findings suggest that BBR is a promising therapeutic agent for mitigating neuroinflammation‐induced cognitive impairment and provide significant evidence for its potential application in treating inflammation‐related cognitive deficits.

## 1. Introduction

Neuroinflammation‐induced cognitive impairment is a complex central nervous system (CNS) disorder, characterized by progressive decline in memory, executive function, and information processing capabilities. These manifestations not only substantially impact patients’ quality of life but also impose considerable socioeconomic burdens [1, 2]. Several studies have indicated that neuroinflammation triggered by surgical trauma, injury, or infection plays a pivotal role in the development of cognitive decline [3–6]. It has been observed that lipopolysaccharide (LPS) administration can temporarily activate microglia and induce long‐term cognitive impairment, indicating the critical role of microglial pro‐inflammatory activation in mediating LPS‐induced cognitive deficits in the mouse model [7, 8]. Moreover, microglial accumulation around cerebral vasculature can be observed as early as 6 h post‐LPS injection, suggesting their rapid role in triggering the neuroinflammatory [9, 10]. Therefore, intracerebroventricular or intrahippocampal LPS injection has been established as a reliable method for modeling cognitive deficits in rodents [11, 12]. Furthermore, the simultaneous microglial activation and alterations in neuronal morphology render the model particularly suitable for investigating inflammation‐related mechanisms in cognitive impairment. Microglia are the primary immune cells in the brain that retain a vigilant presence in healthy states by assuming a ramified shape characterized by elongated processes. They can actively surveil various external environmental stimuli, such as pathogens, cellular debris, and misfolded protein aggregates, all of which were subsequently identified for phagocytic elimination [13]. M1‐type microglia are crucial for exacerbating neuroinflammation. Once activated, microglia undergo homeostatic dysfunction and increase the secretion of interleukin (IL)‐1*β*, IL‐6, nitric oxide (NO), and tumor necrosis factor‐alpha (TNF‐*α*), causing synaptic dysfunction and neuronal death in neuroinflammation‐related cognitive disorders [14, 15]. Several studies have indicated that inhibiting microglial cell activation can substantially ameliorate cognitive deficits [8, 16]. For instance, the immune checkpoint molecule TIM‐3 has been shown to directly regulate microglial phagocytosis and inflammatory responses, playing a central role in amyloid‐*β* deposition and tau pathology [17]. A recent study demonstrated that TREM2 deficiency aggravates *α*‐synuclein‐induced lysosomal deficits in microglia, contributing to cognitive decline in Parkinson’s disease [18]. In addition to these specific regulators, various signaling pathways have been identified as potential targets for improving cognitive function by regulating microglial‐mediated neuroinflammation, including NF‐*κ*B, NLRP3, PPAR‐*γ*, P2X7R, TLR4, cGAS‐STING, and MAPK [19–22].

Berberine (BBR) is a quaternary ammonium isoquinoline alkaloid, which is primarily found in plants belonging to the Berberidaceae, Papaveraceae, Ranunculaceae, and Rutaceae families. It has multiple pharmacological properties, such as antibacterial, antitumor, antiarrhythmic, antigastric ulcer, hypotensive, hypolipidemic, hypoglycemic, anti‐myocardial ischemia, and immunomodulatory effects [23, 24]. Research indicates that BBR enhances neuronal differentiation and improves the survival rates of hippocampal progenitor cells and differentiated neurons [25]. Furthermore, BBR has been found to activate AMP‐activated protein kinase (AMPK), downregulate *β*‐site amyloid precursor protein cleaving enzyme 1, and reduce amyloid‐beta (A*β*) plaques in mouse neuroblastoma cells and primary cultured neurons, thus providing neuroprotective effects [26, 27]. However, whether BBR ameliorates neuroinflammation‐associated cognitive impairment by inhibiting microglial activation‐mediated neuroinflammation in mice remains undetermined. Therefore, this study hypothesized that BBR can alleviate cognitive impairment by modulating microglial‐mediated neuroinflammation. This hypothesis was tested by establishing LPS‐induced neuroinflammatory models in both BV2 cells and mice and evaluating BBR’s therapeutic mechanisms.

This study revealed that BBR inhibits microglial inflammation, suppresses MAPK signaling pathway activation, and improves synaptic function in neurons, mitigating neuroinflammation‐induced cognitive deficits. Our result provides mechanistic evidence supporting the use of BBR as a potential therapeutic agent for inflammation‐related cognitive deficits, highlighting the need for further research into natural compounds for neuroinflammatory disorders.

## 2. Materials and Methods

### 2.1. Animal Model and Sample Collection

Male C57BL/6J mice (8‐week‐old, 18–22 g) were acquired from the Laboratory Animal Center at Southwest Medical University. All *in vivo* analyses followed the Institutional Animal Care and Use Committee at Southwest Medical University (permit number: SYXK 2021‐0264). The mice were housed in well‐ventilated cages at a 12 h light/dark cycle, with ad libitum access to food and water. After a week of acclimatization, mice were randomly divided into four groups (*n* = 14/group): control, LPS, LPS + low BBR (50 mg/kg), and LPS + high BBR (100 mg/kg) groups. The control and LPS group mice were administered a gavage of 0.9% sodium chloride (100 µL) for 21 days. The BBR group mice received a daily gavage of BBR for 21 days. On day 14, mice were anesthetized with alfaxalone (10 mg/kg) *via* intraperitoneal injection and injected with LPS (0.3 µL, 2 mg/mL) in the bilateral hippocampal CA1 regions, specifically at AP + 4.5 mm, ML ± 2.5 mm, and DV −3.0 mm, relative to the bregma in the LPS and LPS + BBR groups. Following the injection, the needle was deliberately retained in the hippocampus bilaterally for 5 min to facilitate the complete absorption of LPS.

### 2.2. Hematoxylin‐Eosin (HE) and Nissl Staining

The HE staining procedure was carried out according to the established protocols [25]. Briefly, mice were perfused with a 4% paraformaldehyde (PFA) solution, and the brain tissue was harvested, fixed in 4% PFA at 4°C for 24 h, sectioned, and then stained. Then, the sections were dehydrated, transparentized, and stained with HE solution (GP1031, Servicebio) or 1% toluidine blue staining solution (GP1052, Servicebio). Images were acquired via a microscope (Olympus, BX63).

### 2.3. Golgi Staining

Golgi staining of the fresh brain tissue sections was performed by following the instructions of the FD rapid Golgi staining kit (PK401, FD NeuroTechnologies, Columbia, MD, USA). Furthermore, the morphology and density of pyramidal neurons and their dendrites within the CA1 region of the mice were assessed using an LSM980 microscope. Moreover, dendritic spines were quantified, and their lengths were assessed via ImageJ software version 6.0. Last, the total dendritic spine density was calculated.

### 2.4. Immunofluorescence and TUNEL Staining

Immunofluorescence staining was conducted according to previously published protocols [28]. Briefly, brain sections were treated overnight at 4°C with primary antibodies against Iba‐1 (1:1000, ab178846, Abcam), PSD95 (1:200, ab13552, Abcam), Synaptophysin (1:500, 4329, CST), iNOS (1:1000, ER1706‐89., Huabio), Arg1 (1:1000, ab91279, Abcam), and NeuN (1:500, 24307, CST). The sections were then washed, probed with either anti‐rabbit IgG H&L Alexa Fluor 488 (1:500, ab150077, Abcam) or goat anti‐mouse IgG H&L Alexa Fluor 594 (1:500, ab150116, Abcam) secondary antibodies, and stained with DAPI (1:300, C1005, Beyotime) to visualize the cell nucleus. For the TUNEL staining process, the hippocampus tissues from different groups were embedded in paraffin, sliced into sections, and stained according to the manufacturer’s protocol (40307ES20, YEASEN). After washing the brain sections with PBS, they were stained with DAPI for 5 min and imaged using an LSM980 microscope, a high‐resolution confocal laser scanning microscope capable of multichannel imaging.

### 2.5. Morris Water Maze (MWM) Test

The MWM test was performed according to standard protocol [28]. Before the assessment, the mice were subjected to adaptive swimming training sessions, which were conducted twice in the morning and twice in the afternoon, with at least 1 h of interval between the two sessions. A spatial exploration test was conducted on the 6th day of the experiment. The exact duration of the experimental subjects’ stay in the target area was meticulously recorded during the test.

### 2.6. Hippocampal Long‐Term Potentiation (LTP) Recordings

LTP was recorded from 350 μm‐thick acute hippocampal slices. The tissues were sliced in ice‐cold artificial cerebrospinal fluid (ACSF), which was continuously oxygenated with carbogen (95% O_2_/5% CO_2_). Subsequently, the slices were allowed to recover in oxygenated ACSF at 35°C for 30 min. Following this recovery period, the slices were transferred to room temperature to achieve equilibration before the electrophysiological recordings [29]. During recording, slices were perfused (1 mL/min) in a submersion chamber and visualized under an Olympus BX51Wi microscope. CA1 pyramidal neurons were patched with borosilicate pipettes (3–5 M*Ω*) containing intracellular solution (in mM: 130 K‐gluconate, 20 KCl, 10 HEPES, 2 MgATP, 4 NaCl, 10 EGTA, pH 7.28). Cells were voltage‐clamped at −70 mV with series resistance variation < 20%. After establishing baseline responses (0.05 Hz, 5 min), LTP was delivered to Schaffer collaterals in CA3 by high‐frequency stimulation (100 Hz, three trains, 10 s intervals). Excitatory postsynaptic currents (EPSCs) were monitored for 40 min post‐induction, and the successful LTP was defined as ≥ 15% potentiation at 30 min. All experiments were conducted at 22–25°C, and the data were acquired using PatchMaster software (HEKA Elektronik).

### 2.7. MTT Assay

The BV2 microglia, an immortalized mouse cell line expressing v‐raf and v‐myc genes (Wuhan Hai Cell Biotechnology Co., Ltd.), were cultured in DMEM/F12 basal medium (Beyotime, Shanghai) augmented with 1% penicillin–streptomycin (ST488, Beyotime) and 10% FBS (C04001‐500, VivaCell) in 5% CO_2_ at 37°C. The cells were then treated with 0–320 μmol/L BBR in serum‐free medium for 24 h, followed by 3 h of treatment with 0.5 mg/mL MTT (M1020, Solarbio). The OD was measured at 570 nm using a microplate reader (Thermo Fisher Scientific, California).

### 2.8. Hoechst Staining

The HT22 mouse hippocampal neuronal cells (Wuhan Hai Cell Biotechnology Co., Ltd. were treated with the conditioned medium from BBR‐treated BV2 cells. Then, CCK‐8 solution (10 μL; A311‐01, Wozem, China) was added to each well for 2 h. The OD was measured using a microplate reader at 450 nm. Furthermore, cell nuclei were also stained using Hoechst (C0003, Beyotime), and images were captured via the LSM980 microscope. ImageJ software was used for further analysis.

### 2.9. Fluorescence Emulsion Bead Phagocytosis Assay

BV2 microglial cells (5 × 10^3^ cells/cm^2^) were seeded onto the coverslips (abs7029, Absin) at 37°C in 5% CO_2_ for 12 h for the phagocytosis assay. Green fluorescent latex beads (FluoresbriteTM carboxy YG 2.0‐micron microspheres) were pretreated in FBS at 37°C for 10 min. Subsequently, 100 µL of DMEM containing latex particles (with 0.01% volume percentage) was added to each well at 37°C for 2 h. After the incubation, the wells were washed three times with PBS buffer to remove the fluorescent latex particles that did not bind to the cells. The cells were then fixed with 4% PFA and treated with anti‐Iba1 primary antibody at 4°C overnight. Finally, the LSM980 microscope was employed to acquire relevant images and evaluate the phagocytic ability of the cells as follows:

 

The phagocytosis of BV2 cells ratio=Number of cells with beads Total number of cells×100%.

### 2.10. Reverse Transcription‐Quantitative Polymerase Chain Reaction (RT‐qPCR) Analysis

The total RNA of hippocampal tissues and BV2 microglial cells was extracted from different groups using TRIzol (REC03 B‐100, Vazyme). The qPCR reaction system (20 µL) comprised 1 µL of cDNA template, 10 µL of 2 × SYBR amplification reagents (Q712‐02, Vazyme), 0.5 µL of 10 mm forward and reverse primers, and 8 µL of deionized water. The relative expression levels of IL‐6, IL‐1*β*, and TNF‐*α* genes were quantified using the 2^−*ΔΔ*CT^ method, and GAPDH was used as the reference for normalization. Table 1 indicates the sequences of primers used for this analysis.

**Table 1:** Sequences of the primers for RT‐qPCR.

Gene name	Forward primer (5′‐3′)	Reverse primer (5′‐3′)
GAPDH	CTGGGCTACACTGAGCACC	AAGTGGTCGTTGAGGGCAATG
IL‐1*β*	TGCCACCTTTTGACAGTGATG	CATCTCGGAGCCTGTAGTGC
IL‐6	CTCCCAACAGACCTGTCTATAC	CCATTGCACAACTCTTTTCTCA
TNF‐*α*	ATGTCTCAGCCTCTTCTCATTC	GCTTGTCACTCGAATTTTGAGA

### 2.11. Western Blot (WB) Analysis

This analysis was carried out following a previously reported study [30]. Briefly, BV2 cells and the isolated hippocampal tissues were lysed in RIPA buffer (P0013B, Beyotime) comprising protease/phosphatase inhibitor mixture (P1005, Beyotime). Then, the samples were treated with PMSF (A610425‐0005, Sangon Biotech) for 30 min on ice. After lysis, the proteins were quantified using the BCA protein assay kit (P0009, Beyotime). Then, equal amounts of protein samples were separated by 10% SDS‐PAGE gel electrophoresis and transferred to PVDF membranes. The target bands were visualized and detected using the Omni‐ECL Femto Light chemiluminescence kit (SQ101L, EpiZyme) by following the manufacturer’s instructions. The Image‐Pro Plus software (Media Cybernetics Company, Rockville, USA) was employed to assess the gray values of the bands. The protein expression levels were calculated relative to GAPDH or actin as an internal reference. The primary antibodies used included: anti‐iNOS (ab49999, 1:1000, Abcam), anti‐Arg1 (ab91279, 1:1000, Abcam), anti‐JNK (#9252, 1:1000, CST), anti‐pJNK (#9255, 1:1000, CST), anti‐ERK1/2 (#4695, 1:1000, CST), anti‐pERK1/2 (#4370, 1:1000, CST), anti‐p38 MAPK (#8690, 1:1000, CST), and anti‐pp38 MAPK (#4511, 1:1000, CST) antibodies. The band intensities of the above antibodies were obtained after standardization with GAPDH or *β*‐tubulin.

### 2.12. Molecular Docking Analysis

Molecular docking analysis was carried out as previously described [31]. The 3D structure of proteins was downloaded from the Protein Data Bank (PDB), and the receptor proteins included MAPK P38 (PDB ID: 3GP0), ERK2 (PDB ID: 4FV0), JNK1 (PDB ID: 2NO3), and JNK2 (PDB ID: 3NPC). The corresponding protein crystal structure was imported into the Maestro system, and the protein preparation was completed using the “Protein Preparation Wizard” function of Schrödinger software. The 3‐D structure of the small molecule ligand BBR was acquired from the PubChem database and processed using the “LigPrep” module of the Schrödinger software suite. Furthermore, hydrogen atoms were added, and bond lengths and angles were corrected using the OPLS‐2005 force field. Moreover, various ionization states, mutual isomers, and stereoisomers of the ligand were generated using the Epik module. Then, the ligands were subjected to molecular docking through the Glide virtual screening workflow. Flexible docking was executed on the designated receptor grid, employing the Extra Precision feature of the Glide module, version 10.1 (2015). The binding free energy of the ligand molecules was then calculated via the Prime MM‐GBSA algorithm using the docked pose retrieved from the Glide algorithm. This module operates based on OPLS_2005 molecular mechanics energy, nonpolar solvation, and the VSGB solvent model. The Prime MM‐GBSA module generates binding free energy using posture viewer files of ligands and receptors (generated after docking). MM‐GBSA analysis uses molecular mechanics and solvent accessibility methods to describe free energy. Last, the PyMol software (version 3.1.0) and BIOVIA Discovery Studio Visualizer (version 2021) software were employed to uncover the details of ligand‐receptor interactions.

### 2.13. Statistical Analysis

The data are presented as mean with standard error of mean (SEM), and n represents the number of independent biological experiments. Statistical analysis was conducted employing GraphPad Prism 10.1.2 (GraphPad Software Inc., USA). One‐way analysis of variance (ANOVA) was utilized for multiple group comparisons, followed by Dunnett’s post hoc test. *p*‐value < 0.05 indicated a statistically significant difference.

## 3. Result

### 3.1. The Effect of BBR on Hippocampal CA1 Tissue Pathology and Apoptosis in the LPS‐Induced POAD Model

Histological analyses via HE and Nissl staining assessed the impact of BBR treatment on neuronal apoptosis. The CA1 region’s neurons in the control group showed abundant cell bodies, regular nuclear shapes, tightly arranged cells, and visible Nissl bodies (Figure 1A,B,D,E). Furthermore, neurons in the LPS‐treated group had shrunken cell bodies, irregular shapes, and substantially reduced Nissl bodies in the cytoplasm (Figure 1A,B,D,E). In comparison to the LPS group, BBR therapy significantly enhanced neuron density and the occurrence of Nissl bodies dose‐dependently, indicating the neuroprotective impact of BBR (Figure 1A,B,E). Furthermore, TUNEL staining was employed to evaluate the impact of BBR on the apoptosis of hippocampal neurons. The results demonstrated a significant rise in TUNEL‐positive cells in the LPS group (Figure 1C,F). In comparison, the LPS + BBR‐H group significantly reduced the number of apoptotic cells, suggesting its efficacy in inhibiting the apoptotic process (Figure 1C,F).

Figure 1The effect of BBR on hippocampal CA1 tissue pathology in the LPS‐induced cognitive deficits model. (A) HE staining and (B) Nissl staining demonstrated histopathological alterations in the hippocampal CA1 region in various treatment groups. Nuclear pyknosis is indicated by blue arrows. (C) Tunnel and DAPI staining were carried out to assess the level of cell apoptosis. (D) Quantitative analysis of the number of neurons in the hippocampal CA1 region of different treatment groups. (E) Quantitative analysis of the number of surviving neurons in the hippocampal CA1 region of different treatment groups. (F) Quantitative analysis of the percentage of apoptotic cells in the hippocampal CA1 region for the different treatment groups. Data are presented as mean ± SEM, with significance levels indicated as  ^∗^ *p*  < 0.05,  ^∗∗^ *p*  < 0.01,  ^∗∗∗^ *p*  < 0.001,  ^∗∗∗∗^ *p*  < 0.0001, ns = no significant difference. Scale bar: 20 μm. (*n* = 6).

**Figure:**
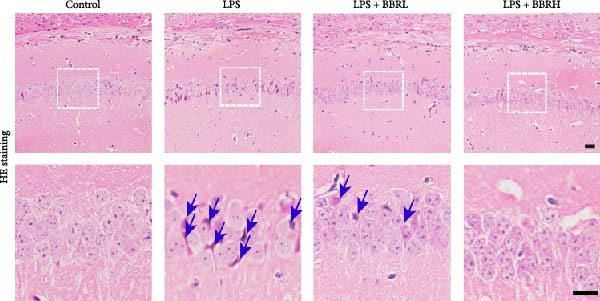
(A)

**Figure:**
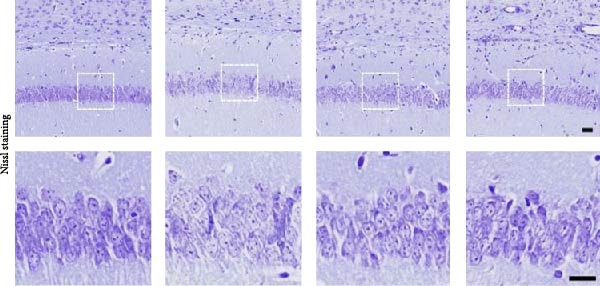
(B)

**Figure:**
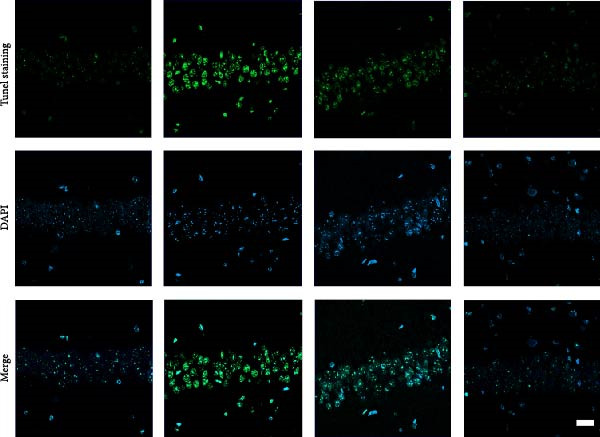
(C)

**Figure:**
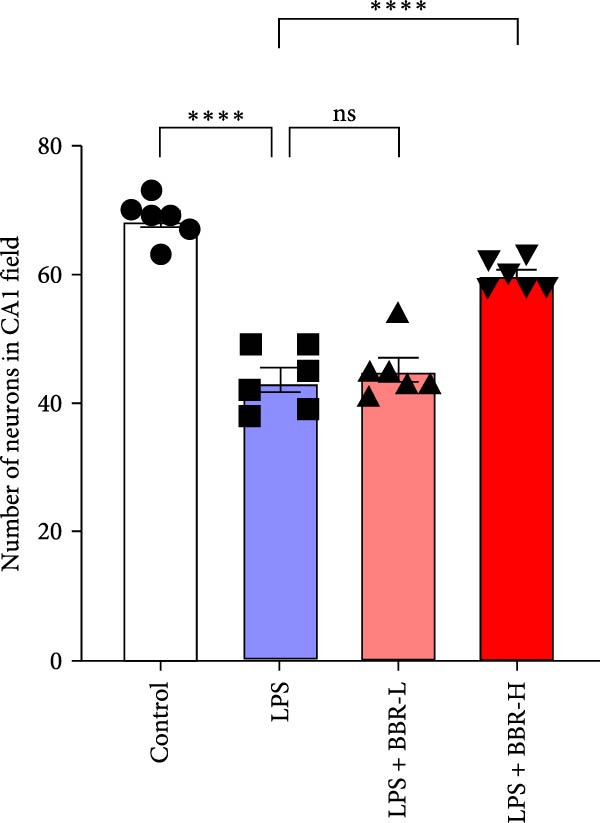
(D)

**Figure:**
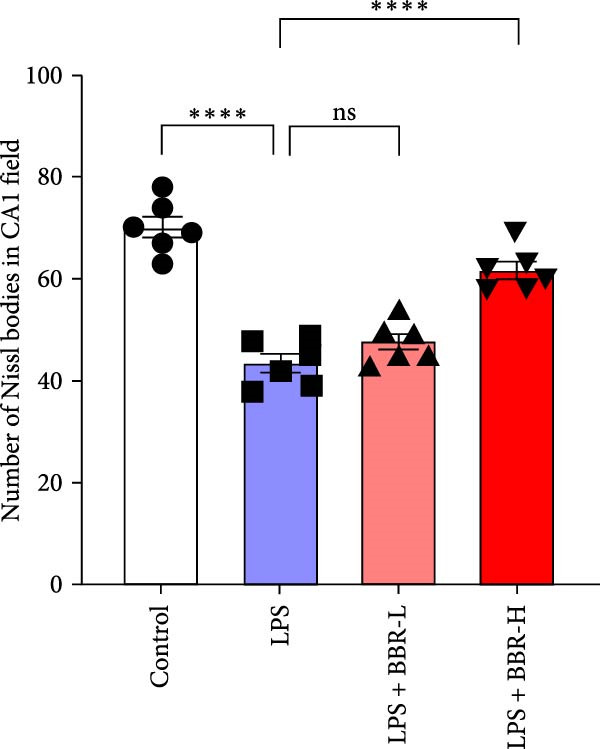
(E)

**Figure:**
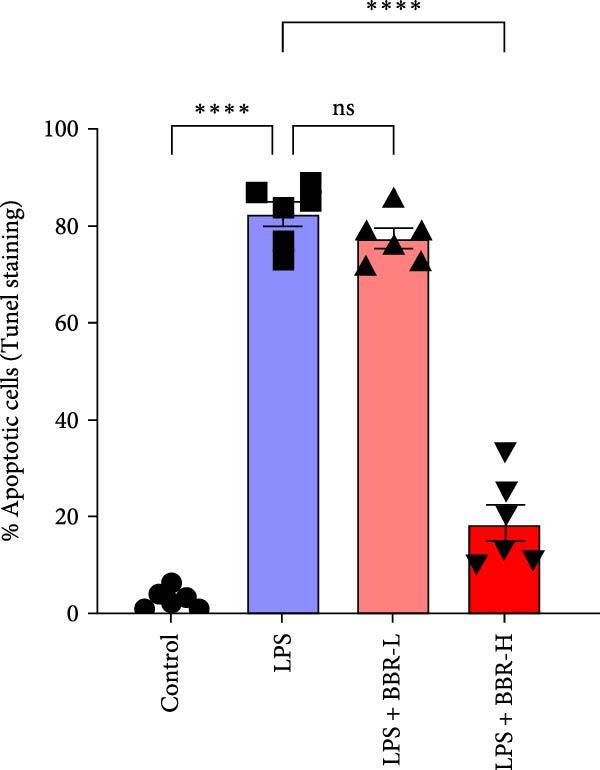
(F)

### 3.2. BBR Enhances Cognitive Function and Electrophysiological Properties in the Hippocampus of LPS‐Induced cognitive Deficit Mice

The MWM assessment was carried out to evaluate the effects of BBR on LPS‐induced cognitive deficits in mice. After training, statistical analysis of the movements revealed that LPS‐treated mice had significantly prolonged escape latencies, indicating the presence of memory deficits (Figure 2C). The LPS + BBR‐H group indicated a statistically significant decrease in escape latency compared to the LPS group. However, there was no significant improvement in the LPS + BBR‐low dose group (Figure 2C). In comparison, the LPS + BBR‐H group mice’s trajectory was concentrated more around the platform, indicating better memory retention ability (Figure 2B). The results demonstrated that 100 mg/kg BBR treatment alleviates cognitive deficits associated with LPS‐induced inflammation. To further examine the functional changes induced by BBR in mitigating cognitive deficits in mice, LTP was evaluated [32]. Whole‐cell patch‐clamp recordings of CA1 pyramidal neurons within acute hippocampal slices were obtained from various groups to investigate LTP. The data revealed a significantly reduced LTP in LPS mice compared to the control group, whereas the LPS + BBR‐H group reversed this effect, improving cognitive deficits associated with neuroinflammation (Figure 3C,D). Moreover, no significant discrepancies in baseline electrophysiological characteristics were observed among the treatment groups (our unpublished data). This finding suggests that the advantageous effects of high‐dose BBR might be specifically associated with the restoration of LTP deficits, rather than modulating the electrophysiological characteristics of the mice.

Figure 2BBR mitigates LPS‐induced learning and memory impairment in mice. (A) Representative trajectories from the open field test demonstrate the exploratory behavior of mice between the four experimental groups. (B) Quantitative analysis of the exploration distance during the initial 5 days of the open field test. (C) Escape latency results from the MWM test for each group. Data are presented as mean ± SEM (*n* = 6/group). Statistical significance is denoted by  ^∗∗∗^ *p*  < 0.001,  ^∗∗∗∗^ *p*  < 0.0001 compared to the control group. ^#^ *p*  < 0.05, ^##^ *p*  < 0.01, ^###^ *p*  < 0.001, ^####^ *p*  < 0.0001 vs. LPS group. ns = no statistically significant difference between the low‐dose administration group and the LPS group.

**Figure:**
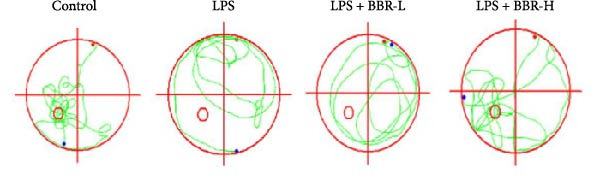
(A)

**Figure:**
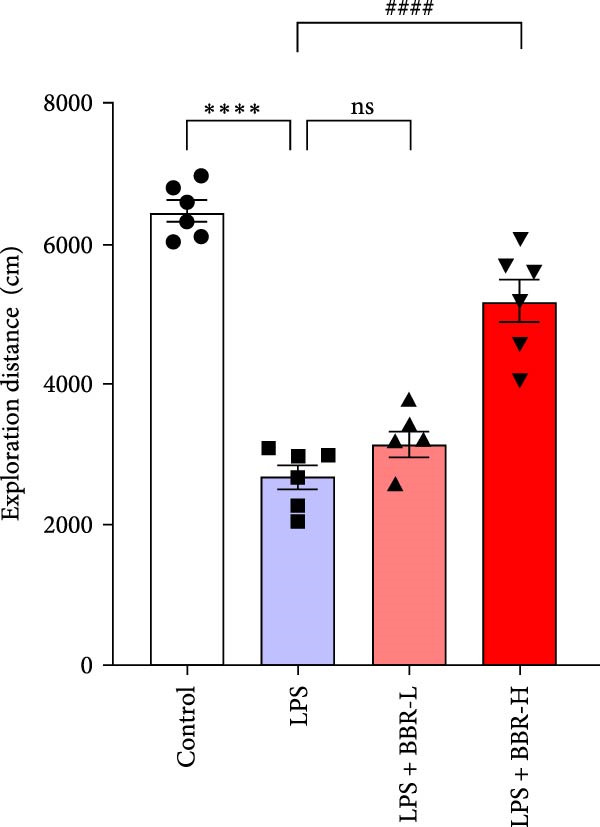
(B)

**Figure:**
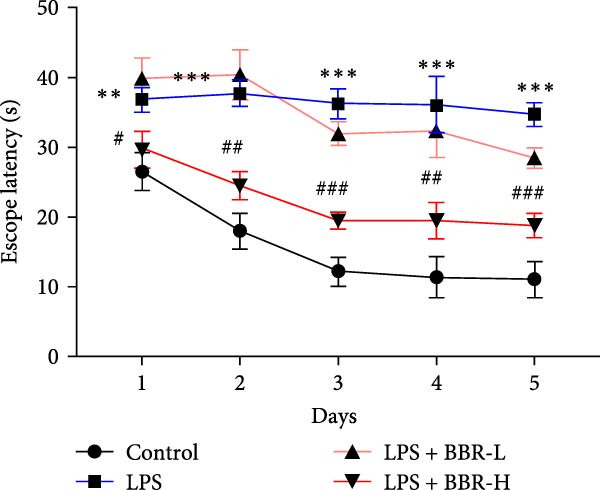
(C)

Figure 3BBR inhibits LTP impairment in LPS‐induced cognitive deficit mice. (A) Representative images of individual EPSCs of the CA1 region after the stimulation of the CA3 region across different treatment groups. (B) Schematic representation of the recording setup in CA1 while stimulating the hippocampal CA3 region. (C) Changes in normalized EPSC size in hippocampal CA1 neurons across each group. Quantitative statistics for EPSCs recorded over 15–30 min are presented in (D). The results are indicated as the mean ± SEM (*n* = 5). Statistical significance is indicated by  ^∗∗^ *p* < 0.01 vs. the control group.  ^∗^ *p* < 0.05 *vs*. LPS group.

**Figure:**
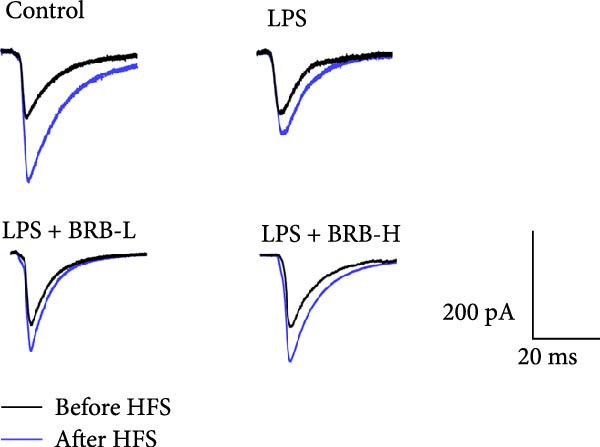
(A)

**Figure:**
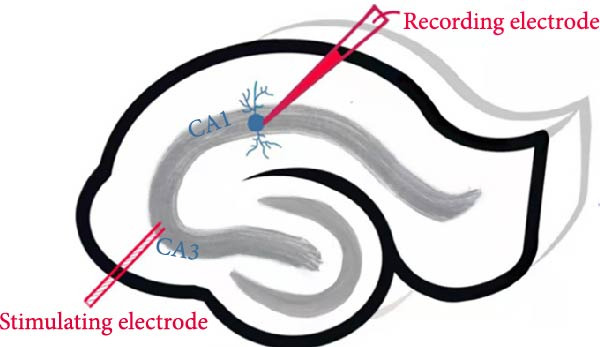
(B)

**Figure:**
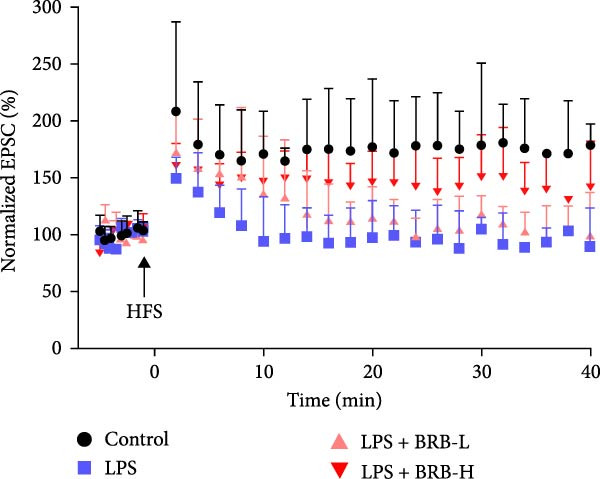
(C)

**Figure:**
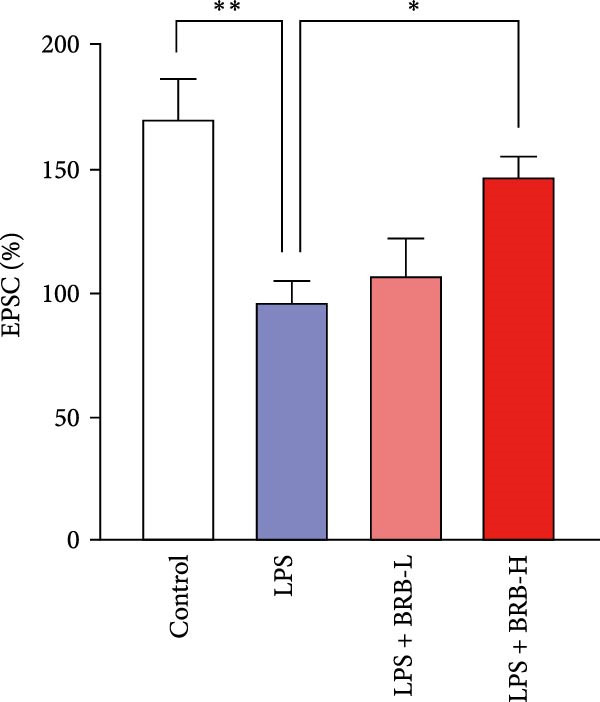
(D)

### 3.3. BBR Modulates Microglial Activation and Polarization

A previous study has demonstrated that BBR promotes a neuroprotective effect by ameliorating cognitive deficits [33]. However, the role of BBR in regulating microglial cell activity and phagocytosis requires additional elucidation. Here, the effects of BBR on modulating microglial activation were assessed, which revealed that high BBR concentration significantly reduced LPS‐induced activation of microglial cells and increased the number of cells in the CA1 hippocampal region (Figure 4A,B). Subsequently, the inducible NO synthase (iNOS) (M1 polarization marker) and arginase‐1 (Arg‐1) (M2 polarization marker) were evaluated to investigate the regulatory effects of BBR on microglial polarization state, providing insights into its potential neuroprotective mechanisms. It was observed that LPS administration significantly upregulated iNOS levels (Figure 5A,C) and downregulated Arg1 levels (Figure 5B,D). These findings suggest that LPS exposure induces a transition in microglial polarization toward the pro‐inflammatory M1 phenotype. Moreover, BBR administration significantly reduced iNOS expression (Figure 5A,C), while concurrently increasing the Arg1‐expressing microglial cell population (Figure 5B,D). The findings suggest that BBR may significantly mitigate neuroinflammatory responses through the modulation of microglial polarization. In addition, the RT‐qPCR analysis was carried out to investigate whether BBR can ameliorate neuroinflammation and subsequently improve cognitive function in mice. The expression levels of inflammatory factors (IL‐6, IL‐1*β*, and TNF‐*α*) in the hippocampus of low BBR dose mice indicated a decreasing trend compared to the LPS group; however, this difference did not reach statistical significance (Figure 5E–G). Moreover, high BBR dosage significantly reduced the mRNA expression levels of IL‐6, IL‐1*β*, and TNF‐*α* in the hippocampus of mice (Figure 5E–G). Due to the restricted effectiveness of low BBR concentration, a high dose of BBR was selected for subsequent analyses to examine its influence on LPS‐induced cognitive impairment. Furthermore, WB experiments were conducted to assess the protein levels of IL‐6, IL‐1*β*, and TNF‐*α* in the hippocampus of the treatment group mice. The results demonstrated that pre‐administration of a high BBR dose significantly suppressed the protein expression of inflammatory factors (Figure 5H,I). Collectively, these findings indicate that a higher BBR dose significantly reduces neuroinflammatory responses by inhibiting microglial M1 polarization and reducing the inflammatory cytokine expression levels in the hippocampal tissues of mice.

Figure 4BBR modulates microglial activation in the LPS‐induced cognitive deficit mice.(A) Representative immunofluorescence staining of right hippocampal sections. Neuronal cells (NeuN, green), microglial cells (Iba1, red), and nuclei (DAPI, blue) were depicted in the control, LPS, and LPS + BBR groups. Scale bar = 50 μm. (B) Quantification of the number of DAPI‐positive cells in the CA1 region. (C) Quantification of the number of Iba1‐positive cells in the CA1 region per 100 μm^2^ in each group. (D) Quantification of the relative fluorescence intensity of Iba1 in the CA1 region of the hippocampus (*n* = 4). Statistical significance was determined using one‐way ANOVA followed by Tukey’s post hoc test. Data are presented as mean ± SEM.  ^∗^ *p*  < 0.05,  ^∗∗^ *p*  < 0.01,  ^∗∗∗^ *p*  < 0.001. ns = no statistically significant difference between the low‐dose administration group and the LPS group.

**Figure:**
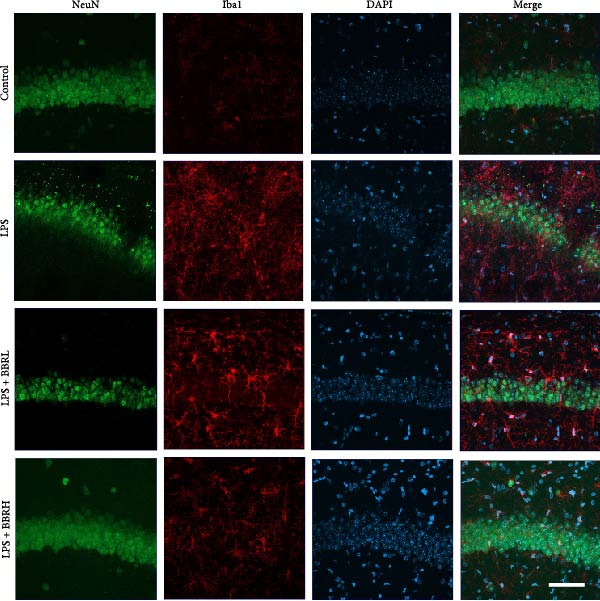
(A)

**Figure:**
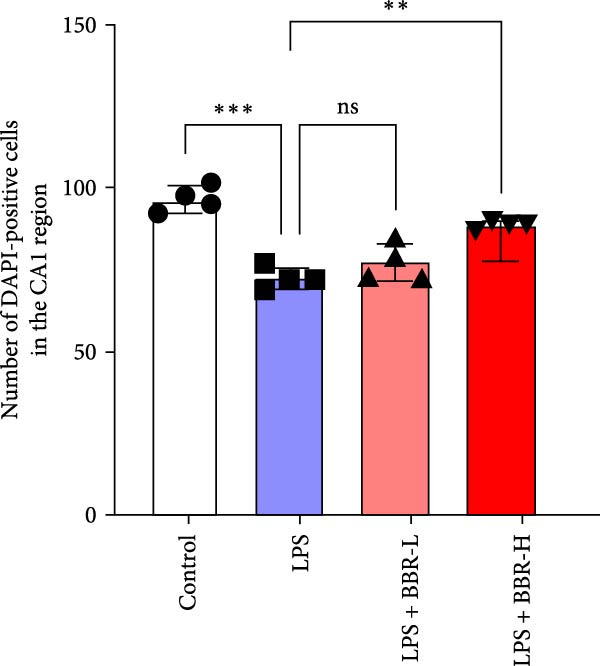
(B)

**Figure:**
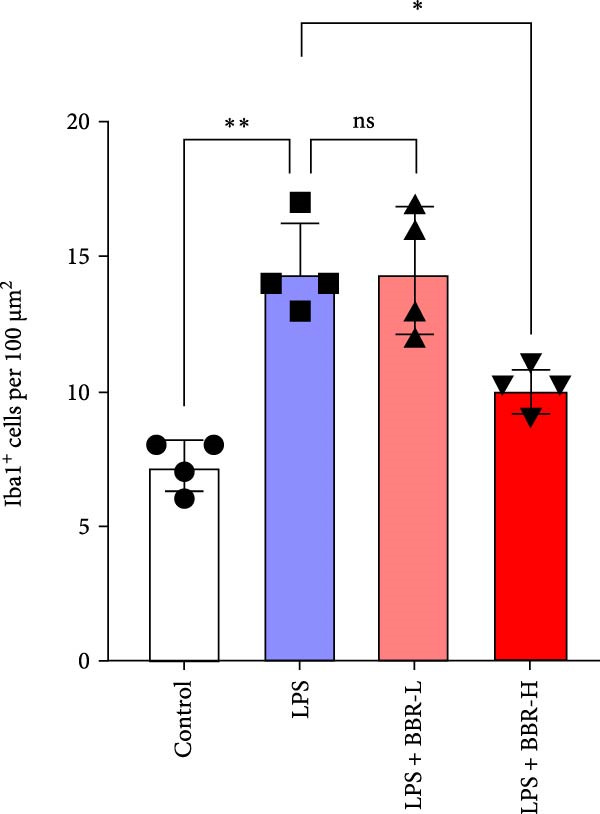
(C)

**Figure:**
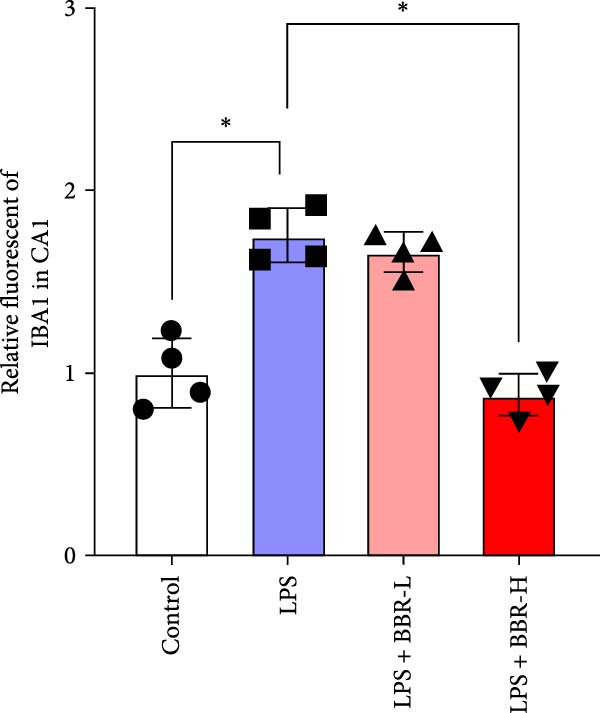
(D)

Figure 5BBR alleviated neuroinflammation and modulated microglial polarization in the LPS‐induced POAD model. Immunofluorescence staining for iNOS (green, A) and Arg1 (green, B) was performed in the hippocampus. Microglial cells were stained for Iba1 (red, A, B), and nuclei were stained with DAPI (blue). Scale bar = 10 μm. Quantification of the relative number of Iba1‐positive cells expressing iNOS (C) or Arg1 (D) in the CA1 region of the hippocampus is shown. The relative mRNA expression levels of TNF‐*α* (E), IL‐6 (F), and IL‐1*β* (G) were measured in the control, LPS, and LPS + BBR (20 and 50 mg/kg) groups. (H‐I) WB analysis was performed to assess the protein expression of Iba‐1, IL‐6, and IL‐1*β* in the hippocampus of mice in each group (*n* = 6). Statistical significance was determined using one‐way ANOVA followed by Tukey’s post hoc test. Data are presented as mean ± SEM.  ^∗^ *p* < 0.05,  ^∗∗^ *p* < 0.01,  ^∗∗∗^ *p* < 0.001,  ^∗∗∗∗^ *p* < 0.0001.

**Figure:**
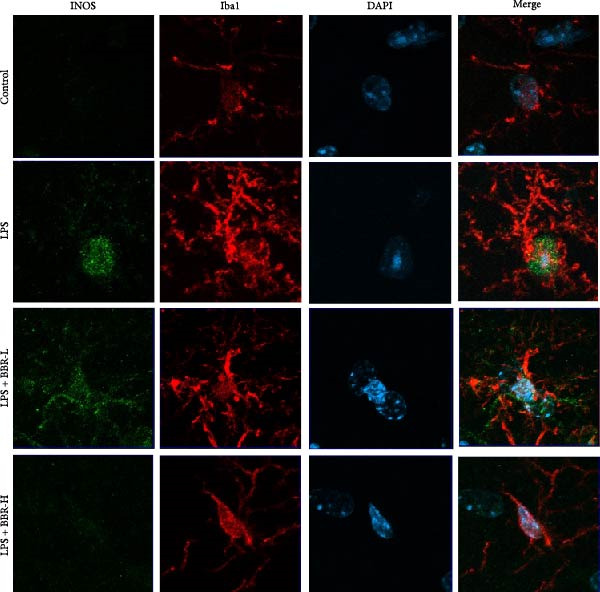
(A)

**Figure:**
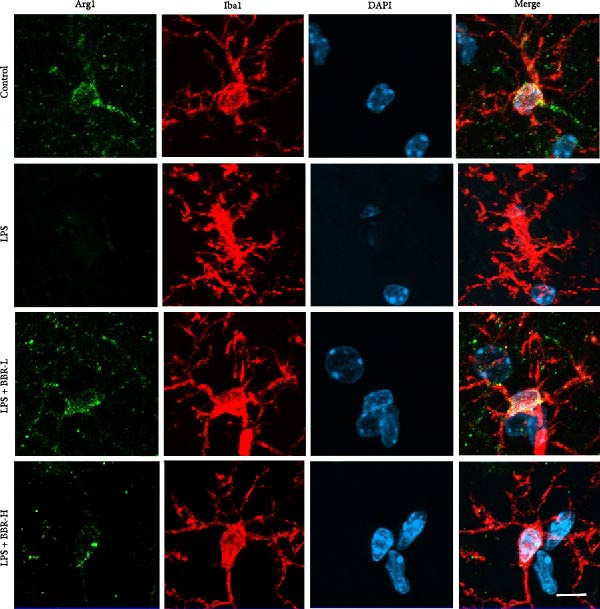
(B)

**Figure:**
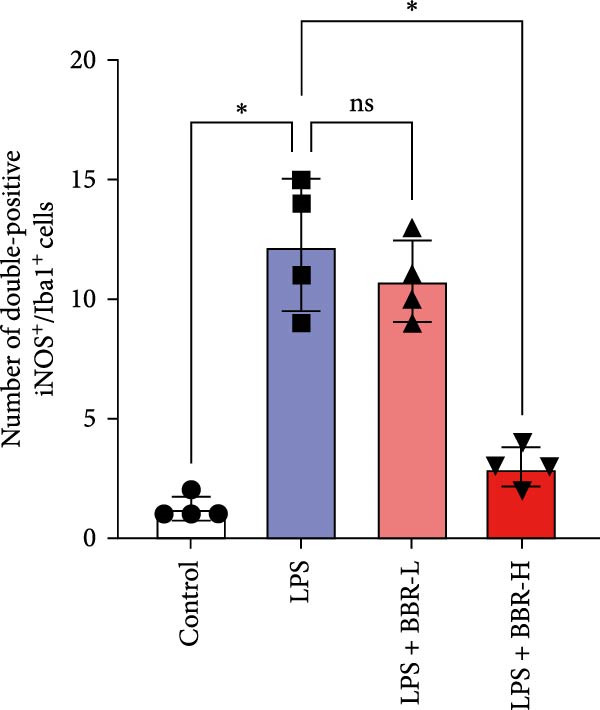
(C)

**Figure:**
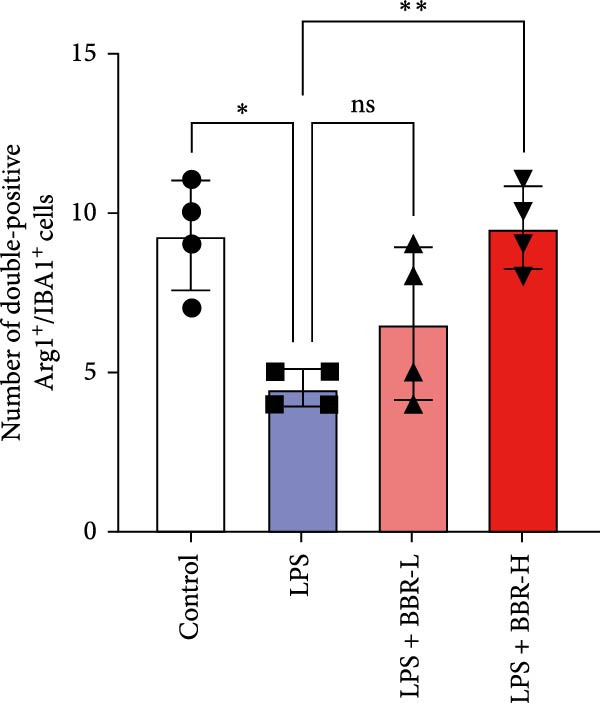
(D)

**Figure:**
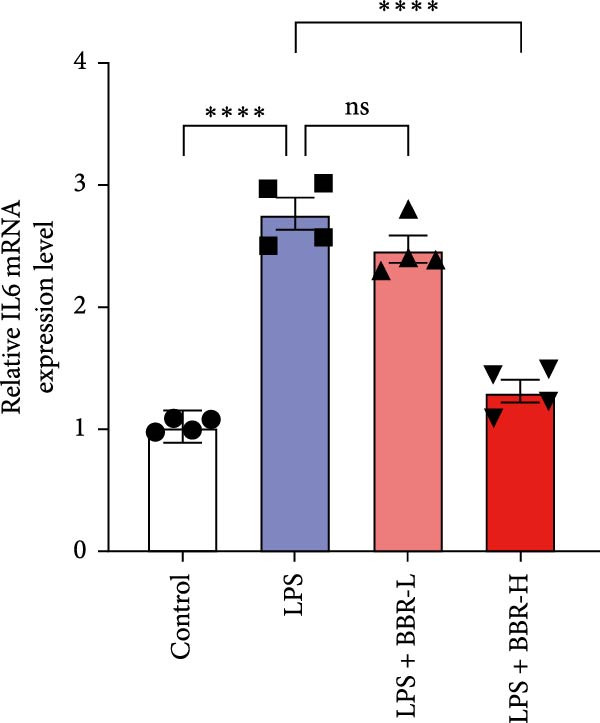
(E)

**Figure:**
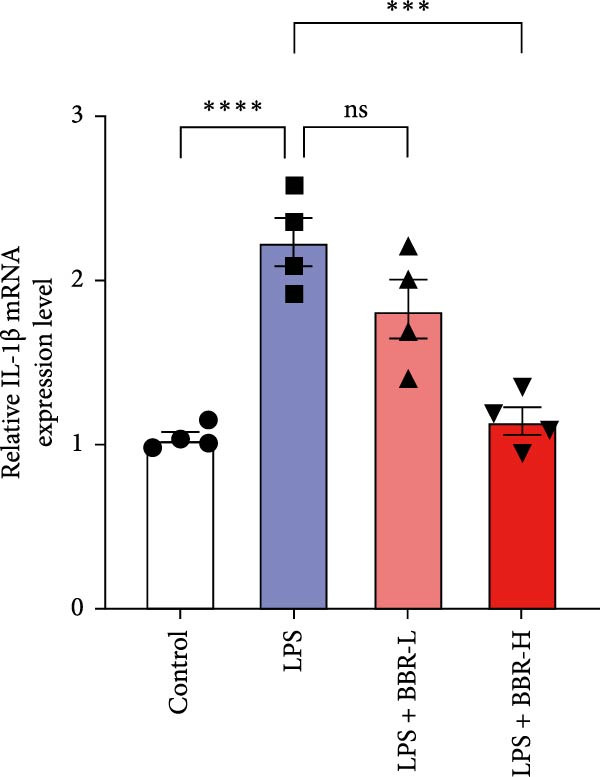
(F)

**Figure:**
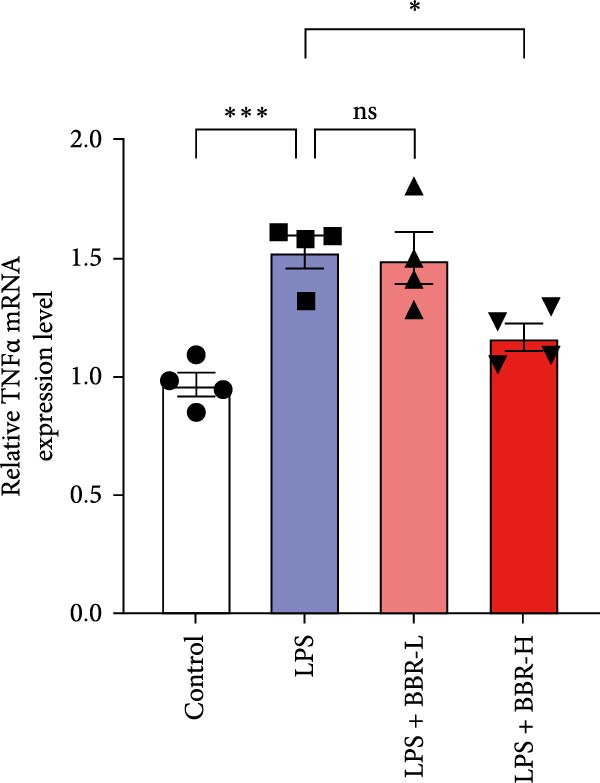
(G)

**Figure:**
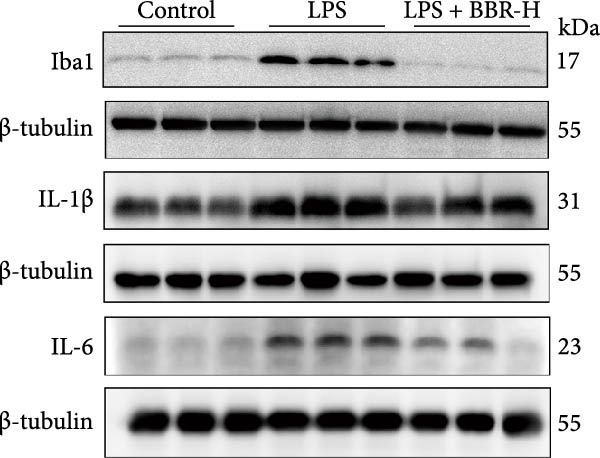
(H)

**Figure:**
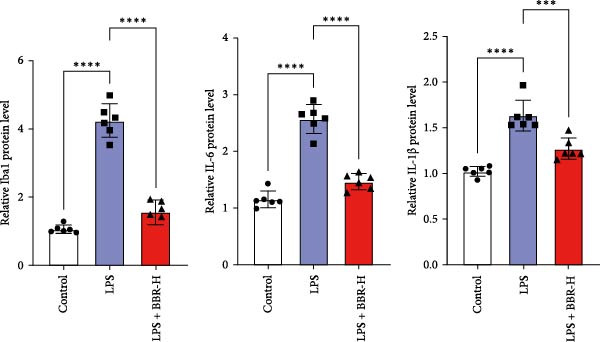
(I)

### 3.4. BBR Treatment Ameliorates LPS‐Induced Dendritic Spine Loss in the Hippocampus

To determine if BBR protects against LPS‐induced synaptic deficits, we performed Golgi staining to assess dendritic complexity and spine density. We found that LPS administration significantly reduced both the dendritic spine density and the complexity of dendritic arbors (as measured by Sholl analysis) in the hippocampus (Figure 6A–C). Conversely, high‐dose BBR treatment effectively restored these parameters in the CA1 region (Figure 6A–C).

Figure 6BBR alleviates the reduced dendritic spine density and synaptic proteins induced by LPS‐induced cognitive deficits in mice. (A) Representative images of dendritic spines in the CA1 region of the hippocampus were obtained using Golgi staining. Quantification of spine density (B) and mushroom spine density (C) in each group. Scale bar = 5 μm. (D) WB detection of retinal PSD95 and synaptophysin protein expression changes across groups. Statistical results for relative protein expression of PSD95 (E) and synaptophysin (F). β‐tubulin was used as a loading control. (G) Immunofluorescence staining of synaptophysin (red) and PSD95 (green) proteins from each group. Nuclei were counterstained with DAPI (blue). Scale bar = 20 μm. Quantification of the mean fluorescence intensity of PSD95 (H) and synaptophysin (I). Data are presented as mean ± SEM.  ^∗^ *p*  < 0.05,  ^∗∗^ *p*  < 0.01,  ^∗∗∗^ *p*  < 0.001.

**Figure:**
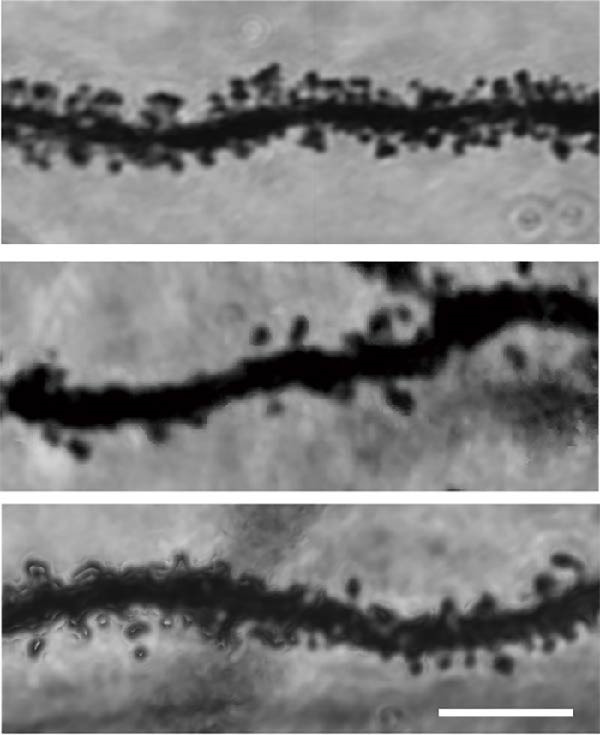
(A)

**Figure:**
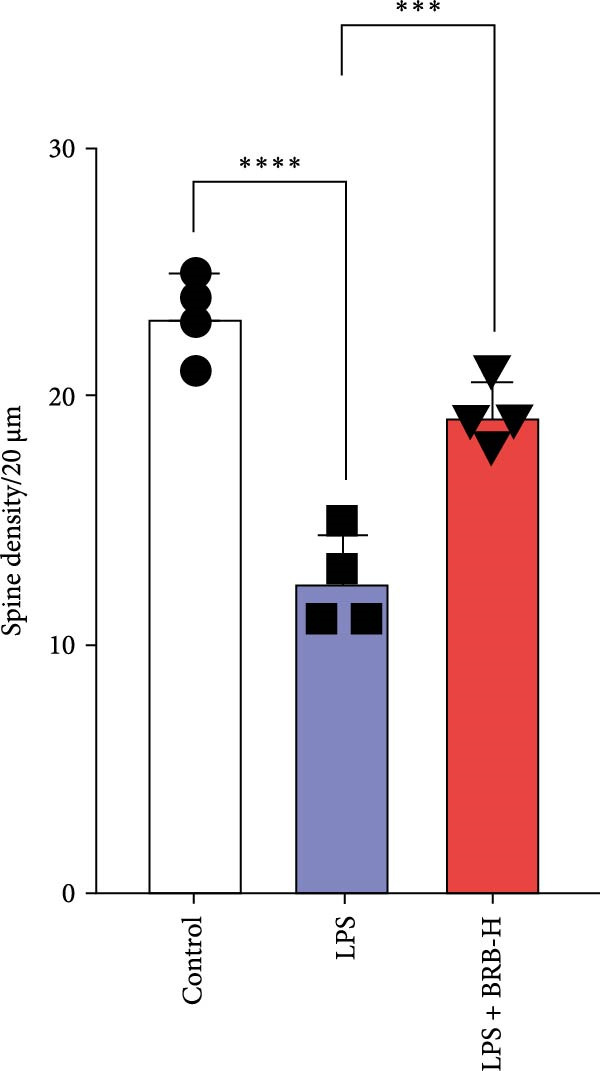
(B)

**Figure:**
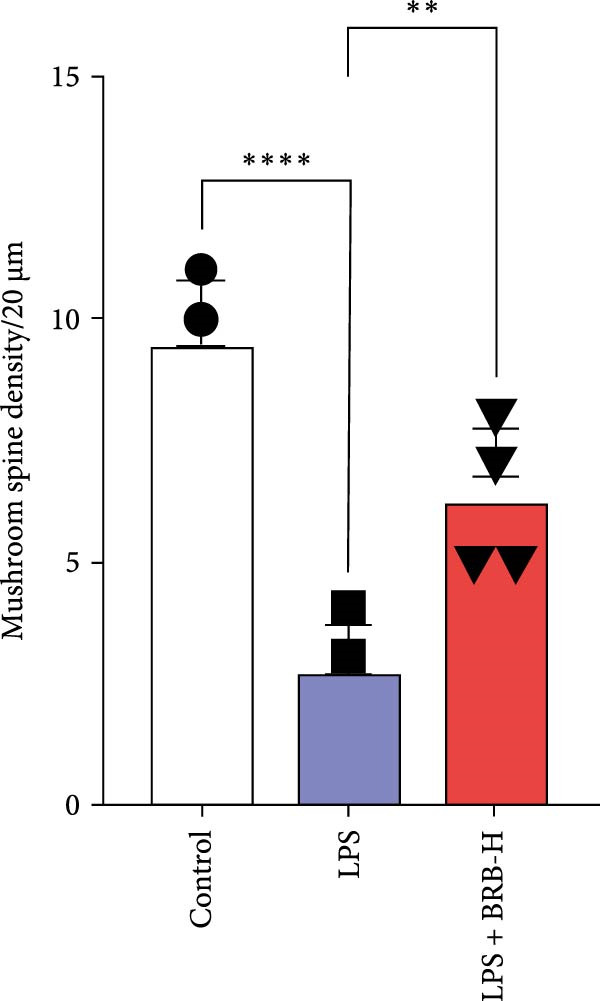
(C)

**Figure:**
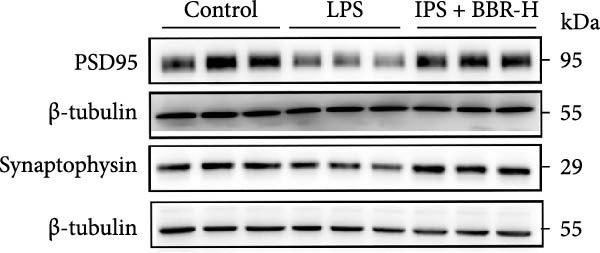
(D)

**Figure:**
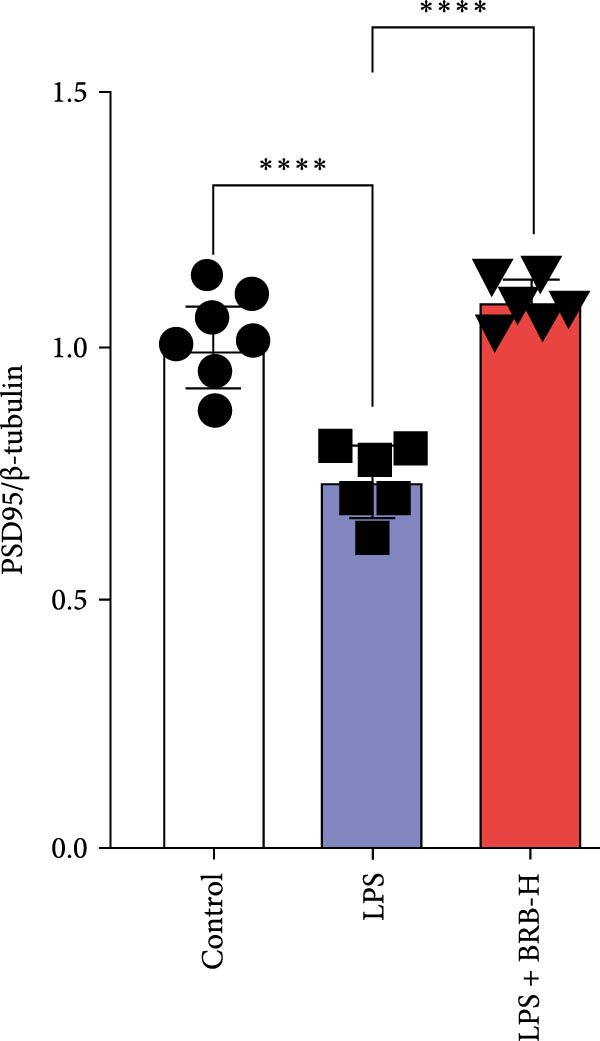
(E)

**Figure:**
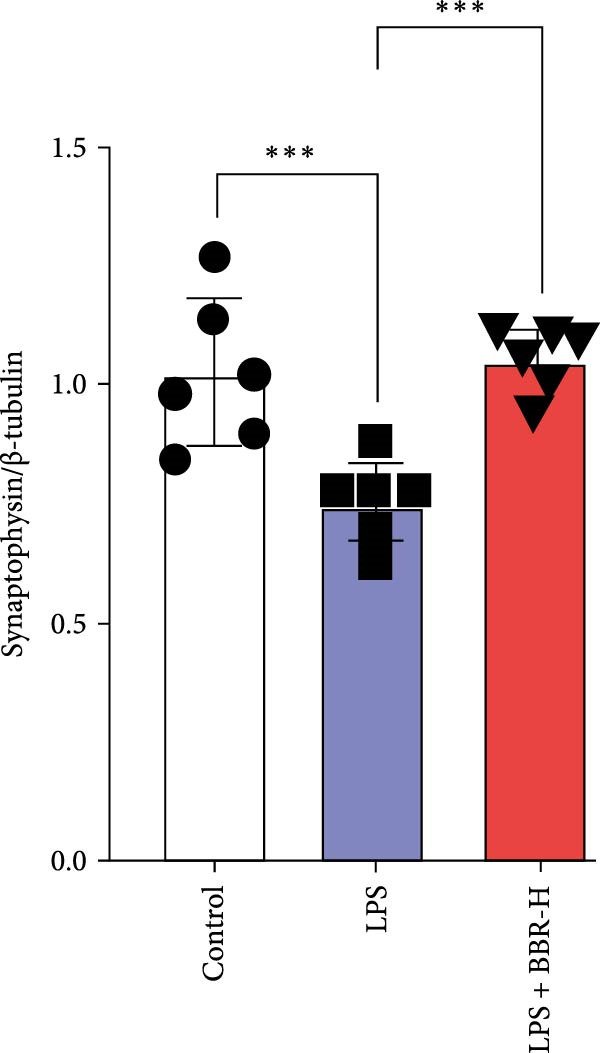
(F)

**Figure:**
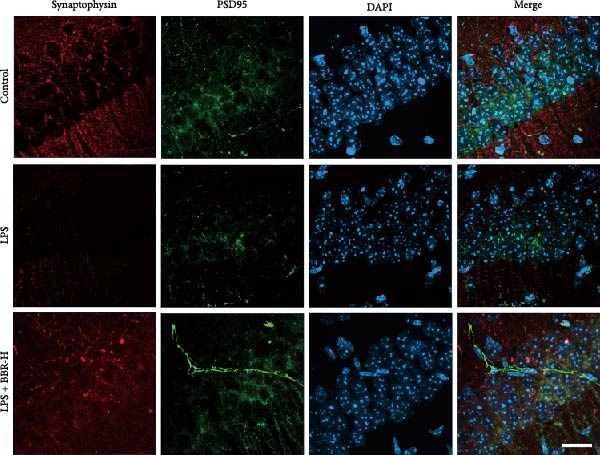
(G)

**Figure:**
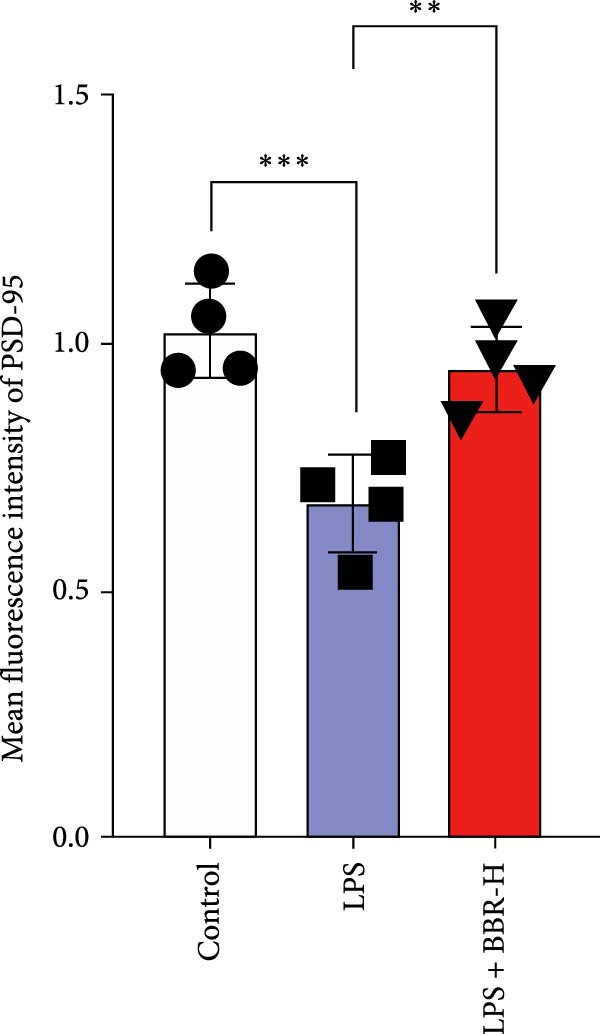
(H)

**Figure:**
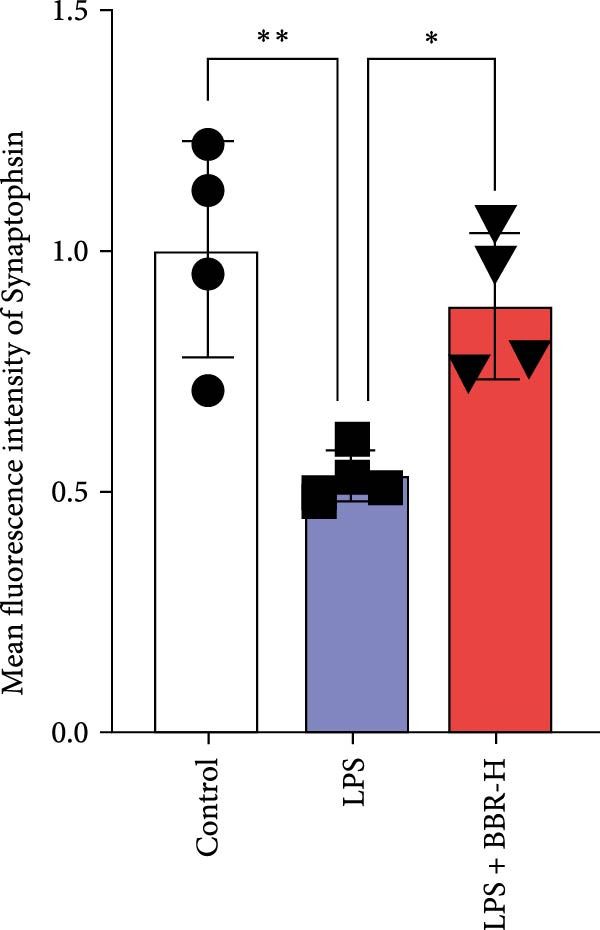
(I)

Dendrites are fundamental structural units that facilitate the transfer of information between neurons. Alterations in dendrite numbers, structure of dendritic spines, postsynaptic density (PSD), and spine morphology can change dendritic structural plasticity [34–36]. To assess whether BBR improves the dendritic structural plasticity of hippocampal neurons across various treatment groups, Golgi staining was carried out to assess the density of dendritic spines. The results indicated that the density and number of intersections of dendritic spines in LPS‐treated mice were significantly decreased (Figure 6A–C, *p*  < 0.05). Whereas high‐BBR treatment significantly increased these parameters in the CA1 hippocampal region (Figure 6A–C). Furthermore, to further investigate whether the reduction in dendritic spines was associated with a decrease in synaptic proteins, the expression levels of PSD‐95 (PSD‐95) and synaptophysin in the hippocampus were assessed *via* immunofluorescence and WB analyses. It was observed that compared to the control group, LPS treatment significantly reduced PSD‐95 and synaptophysin protein expression levels (Figure 6D–F). Furthermore, immunofluorescence analysis showed that LPS administration significantly reduced synaptophysin and PSD‐95 levels in the hippocampal region, which were reversed by BBR treatment (Figure 6G–I). These findings demonstrated that LPS treatment significantly reduced dendritic spine density and synaptic protein expression in the hippocampus, whereas high‐concentration BBR treatment effectively restored these morphological and molecular changes. The results highlight the potential of BBR in mitigating LPS‐induced synaptic damage and preserving hippocampal integrity.

### 3.5. BBR Attenuates LPS‐Induced Cognitive Deficits by Inhibiting Mitogen‐Activated Protein Kinase (MAPK) Signaling Pathway

Research indicates that the MAPK signaling pathway is associated with microglia‐mediated neuroinflammation [37]. Several key proteins were observed in this signaling pathway, including extracellular signal‐regulated kinase (ERK), c‐Jun N‐terminal kinase (JNK), and p38 MAPK. These kinases regulate the expression of critical inflammatory mediators, such as iNOS, IL‐6, and TNF‐*α*, directly impacting the neuroinflammatory pathway [38]. To examine the neuroprotective mechanisms of BBR, particularly its potential anti‐inflammatory effects via microglial modulation, WB analysis was carried out to evaluate the protein expression levels of key signaling molecules, specifically JNK, ERK1/2, and p38 MAPK, across various treatment groups. The findings revealed that BBR significantly attenuated the LPS‐induced elevations in the relative protein expression of pJNK, pERK1/2, and pp38 MAPK (Figure 7A–D). These results suggest that BBR suppresses the LPS‐induced cognitive deficits by modulating the MAPK signaling pathways.

Figure 7BBR concentration‐dependently inhibits LPS‐induced activation of the MAPK signaling pathway. (A) Protein expression levels of phosphorylated JNK (p‐JNK, Thr183/Tyr185), total JNK, phosphorylated ERK1/2 (p‐ERK1/2, Thr202/Tyr204), total ERK1/2, phosphorylated p38 MAPK (p‐p38 MAPK, Thr180/Tyr182), and total p38 MAPK were examined by Western blot in each group (*n* = 6). (B–D) Quantitative analysis of the ratio of phosphorylated protein to total protein expression for JNK (B), ERK (C), and p38 MAPK (D).  ^∗∗^ *p*  < 0.01,  ^∗∗∗^ *p*  < 0.001,  ^∗∗∗∗^ *p*  < 0.0001.

**Figure:**
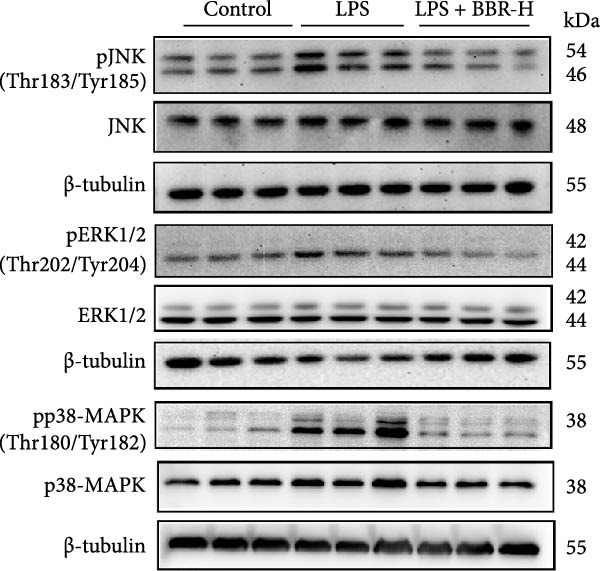
(A)

**Figure:**
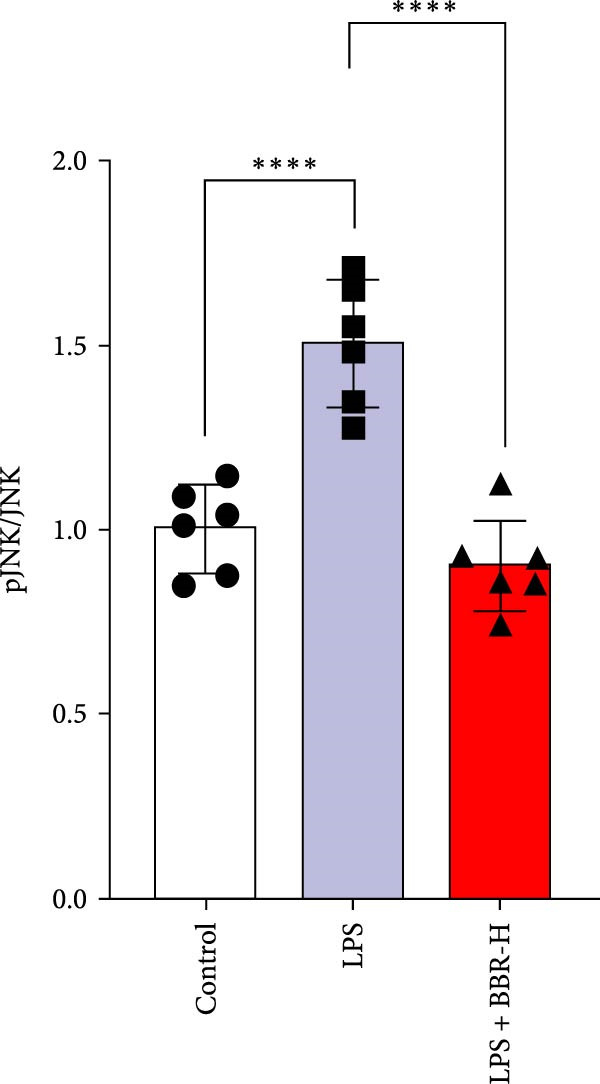
(B)

**Figure:**
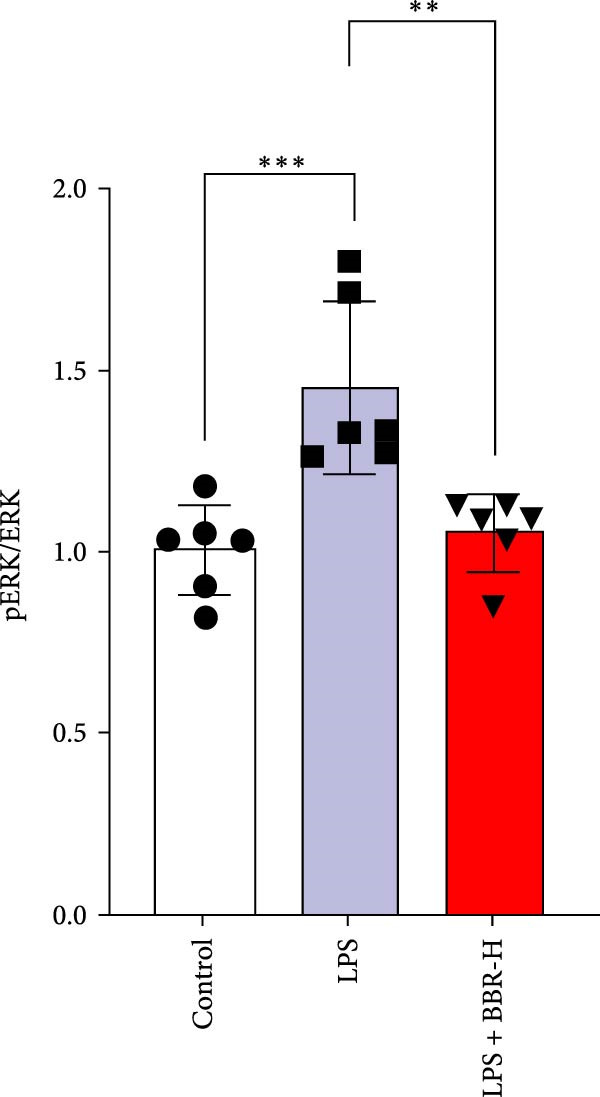
(C)

**Figure:**
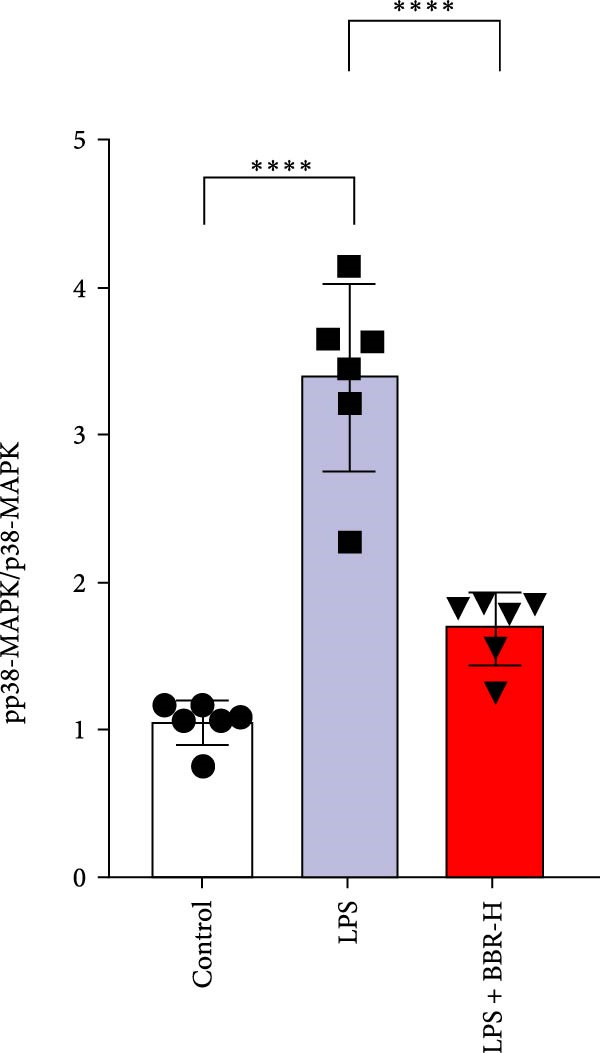
(D)

### 3.6. BBR Enhances Neuronal Survival by Improving the Phagocytic Ability of LPS‐Stimulated BV2 Microglial Cells

To determine whether BBR mitigates cognitive deficits by targeting microglia, we cultured HT22 hippocampal neuronal cells with LPS‐treated BV2 microglia in conditioned medium, either with or without BBR, and assessed neuronal survival through Hoechst staining. The results showed that HT22 cells with the conditioned medium from LPS‐induced microglia promoted cell apoptosis and increased mean fluorescence intensity. This effect was substantially reversed after incubation with a BBR‐treated BV2‐conditioned medium, which was concentration‐dependent (Figure 8A–C). To evaluate whether BBR affects the phagocytosis of BV2 microglia and exerts an anti‐inflammatory effect, BV2 microglia were stimulated with LPS in the presence of fine phagocytic fluorescent beads. The cells were then exposed to various BBR concentrations (10, 20, 40, 80, 160, and 320–μmol/L) for 2 h to determine their potential cytotoxic effects *via* the MTT assay. The results indicated that < 80 µg/mL BBR had no impact on the viability of BV2 cells, regardless of LPS treatment (our unpublished data). Therefore, 40 and 60 µg/mL BBR were selected for investigating the anti‐inflammatory impact of BBR on LPS‐induced BV2 cells. LPS induces M1 microglial polarization. Furthermore, LPS‐stimulated BV2 microglial cells indicated significant morphological alterations, including an increase in cell body size and an extension of cellular processes toward the poles, compared to the control group (Figure 8D). However, BBR treatment ameliorated LPS‐induced morphological alterations in a concentration‐dependent manner (Figure 8E). Moreover, the fluorescence emulsion bead phagocytosis assay demonstrated that BV2 cells’ phagocytic activity was significantly enhanced following LPS stimulation (Figure 8F,G), which was reduced after BBR treatment, concentration‐dependently (Figure 8F,G). Subsequently, the number of beads engulfed by individual BV2 cells in each group was quantified, which significantly increased after LPS stimulation compared to the control group. Compared to the LPS group, the various BBR doses reduced the number of beads within BV2 cells, demonstrating statistically significant differences (Figure 8F,G). These results suggest that BBR effectively inhibits the LPS‐induced phagocytic activity in the BV2 cells.

Figure 8BBR protects against neuronal damage by inhibiting microglial phagocytosis. (A) The Hoechst staining of HT22 cells cultured with LPS or LPS + BBR‐treated BV2 cells conditioned medium for 24 h. (B) Quantitative results of the apoptosis rate and average fluorescence intensity (C) of HT22 cells in different groups. (D) BV2 cells were stimulated by LPS with (40 or 60 μM/L) BBR or without BBR for 24 h and then co‐cultured with green fluorescent latex beads for 2 h. Representative image of BV2 microglia immunolabeled with anti‐Iba1 (red) antibody and nuclei stained with DAPI (blue), *n* = 6, Scale bar = 10 μm. (E) The BV2 cell body diameter was quantified with ImageJ software. (F) The phagocytosis of BV2 cells was quantified as the proportion of cells attached to the beads (proportion of microglia involved in phagocytosis). (G) The number of phagocytized beads by each cell was precisely quantified. Statistical significance was determined using a one‐way ANOVA with Tukey’s post hoc test.  ^∗^ *p*  < 0.05,  ^∗∗^ *p*  < 0.01,  ^∗∗∗^ *p*  < 0.001,  ^∗∗∗∗^ *p* < 0.0001.

**Figure:**
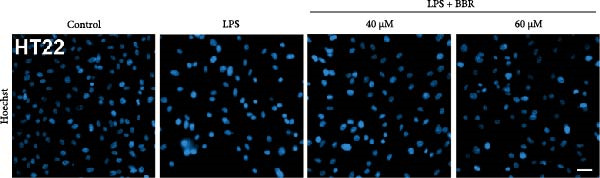
(A)

**Figure:**
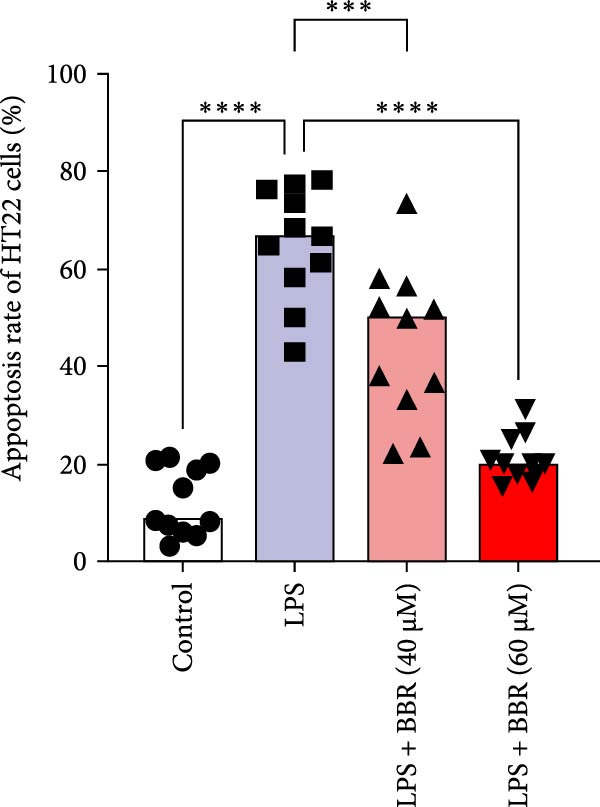
(B)

**Figure:**
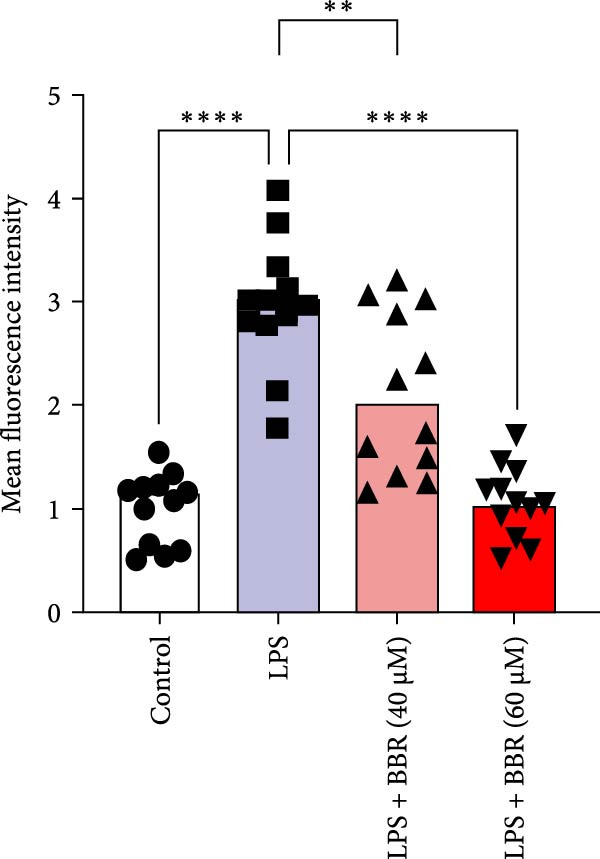
(C)

**Figure:**
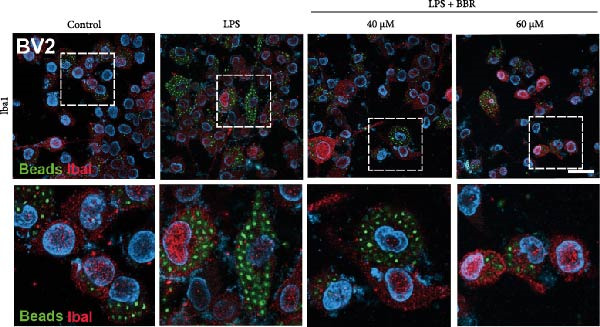
(D)

**Figure:**
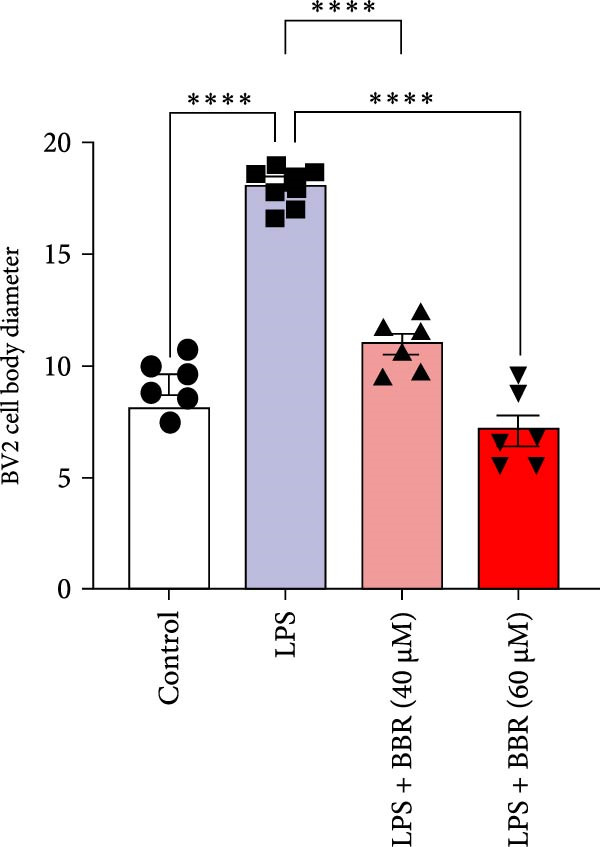
(E)

**Figure:**
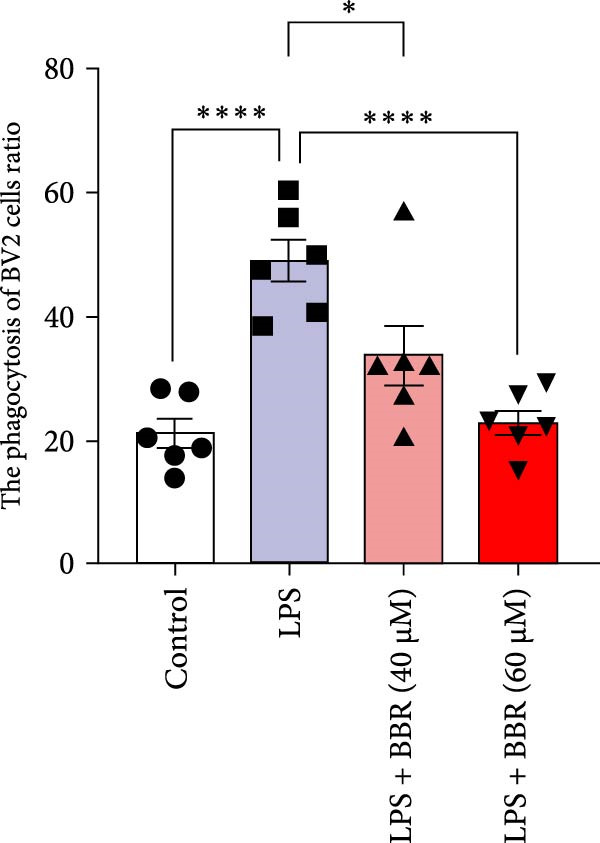
(F)

**Figure:**
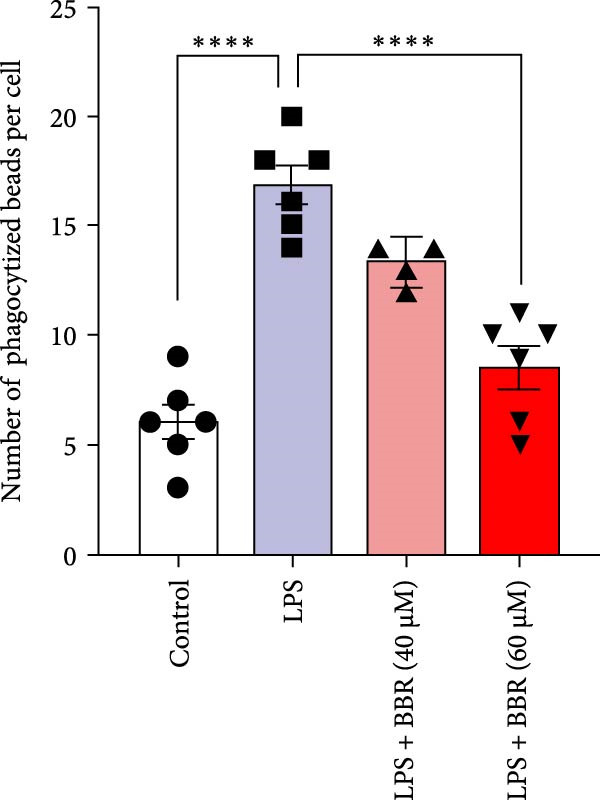
(G)

### 3.7. BBR Attenuates the LPS‐Induced pro‐Inflammatory Cytokine Expression in BV2 Cells and Inhibits the Activation of the MAPK Signaling

To further investigate how BBR influences neuronal viability and apoptosis via microglial polarization, WB analysis was performed to assess the protein levels of M1 polarization markers (iNOS, IL‐6) and M2 polarization markers (Arg1) in various treatment groups. The results indicated that LPS treatment elevated the protein expression levels of iNOS and IL‐6 in BV2 cells, indicating the activation of M1‐type microglia (Figure 9A–C). Furthermore, 40 and 60 μM BBR reduced iNOS and IL‐6 levels, suggesting that BBR effectively attenuates LPS‐induced inflammation and microglial activation (Figure 9A–C). Similarly, it was observed that LPS treatment significantly reduced Arg1 expression, while BBR inhibited this effect (Figure 9D). The experimental results indicated that BBR inhibits LPS‐induced activation of BV‐2 microglia into the pro‐inflammatory M1 phenotype and facilitates a transition to the anti‐inflammatory M2 phenotype. The literature suggests that M1 microglial activation promotes neuronal damage primarily by releasing pro‐inflammatory cytokines such as IL‐6, NO, IL‐1*β*, and TNF‐*α* [38, 39]. To assess the impact of BBR on the expression of pro‐inflammatory cytokine genes in microglial cells, qRT‐PCR analysis was performed, which revealed that the relative mRNA levels of IL‐1*β*, IL‐6, and TNF‐*α* were significantly elevated after LPS stimulation. These data demonstrate that BBR treatment significantly decreased the expression of the aforementioned inflammatory factors (Figure 9E–G). WB data further confirmed that LPS‐stimulated cells had significantly increased JNK, ERK, and p38 MAPK phosphorylation levels compared to the control group, whereas BBR treatment dramatically reduced these levels (Figure 10). These findings indicate that BBR dramatically reduces microglial inflammatory responses by suppressing the MAPK signaling pathway, thus conferring neuroprotective effects.

Figure 9BBR suppressed inflammatory factors and iNOS activity in BV2 microglial cells and increased ARG1 expression. (A–D) WB analysis of IL6, iNOS, and Arg1 protein expression in BV2 cells in the control, LPS, LPS + BBR (40 μM), and LPS + BBR (60 μM) groups. (E–G) Effect of BBR on LPS‐induced inflammatory factors secretions in BV2 cells. The expression levels of inflammatory factors, including IL‐6 (E), IL‐1β (F), and TNF‐α (G), were detected by qRT‐PCR The data were expressed after being normalized to GAPDH (*n* = 6).  ^∗^ *p* < 0.05,  ^∗∗^ *p* < 0.01,  ^∗∗∗^ *p* < 0.001, ^∗∗∗∗^ *p* < 0.001.

**Figure:**
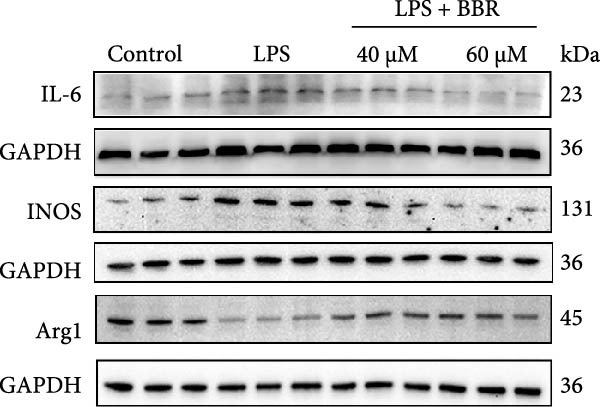
(A)

**Figure:**
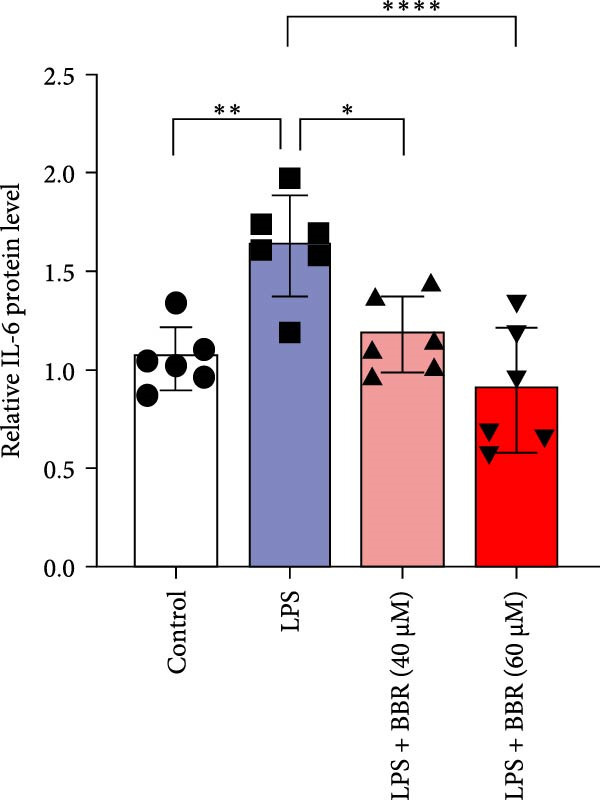
(B)

**Figure:**
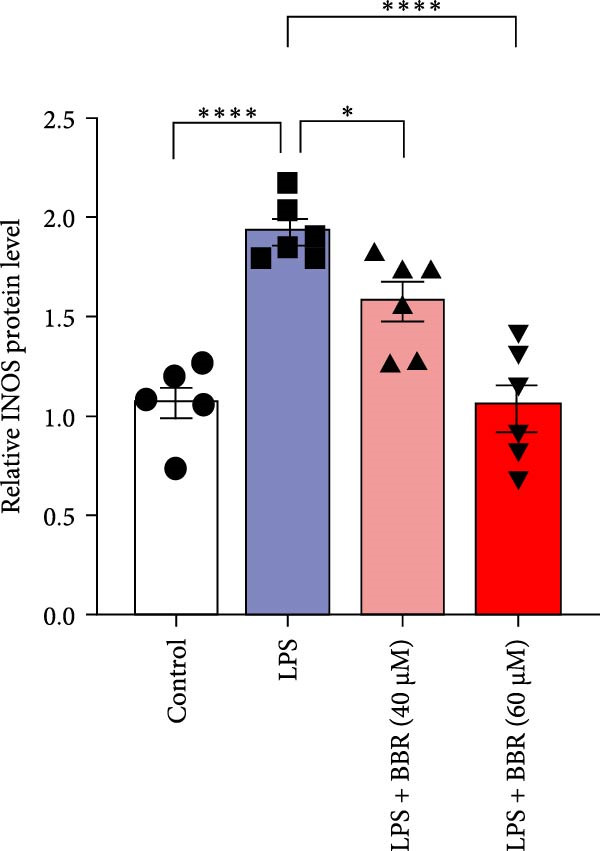
(C)

**Figure:**
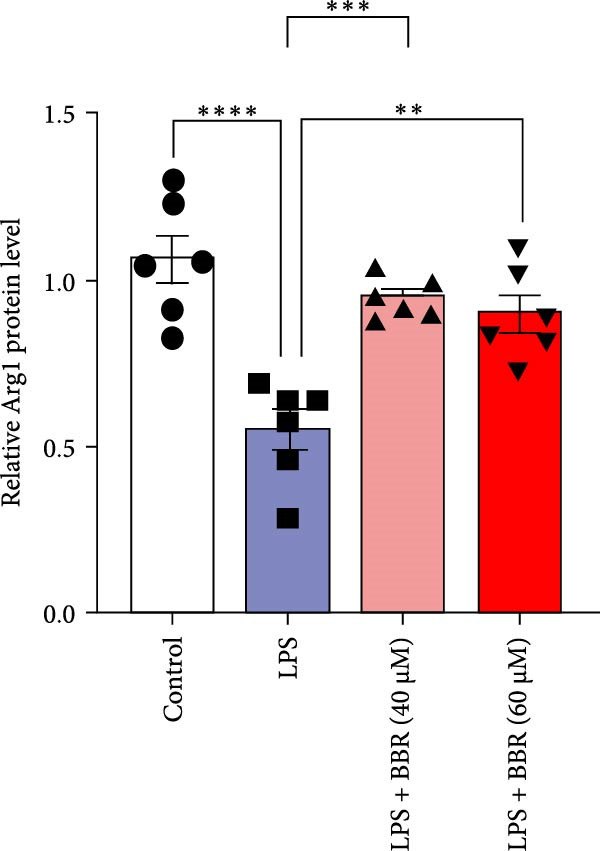
(D)

**Figure:**
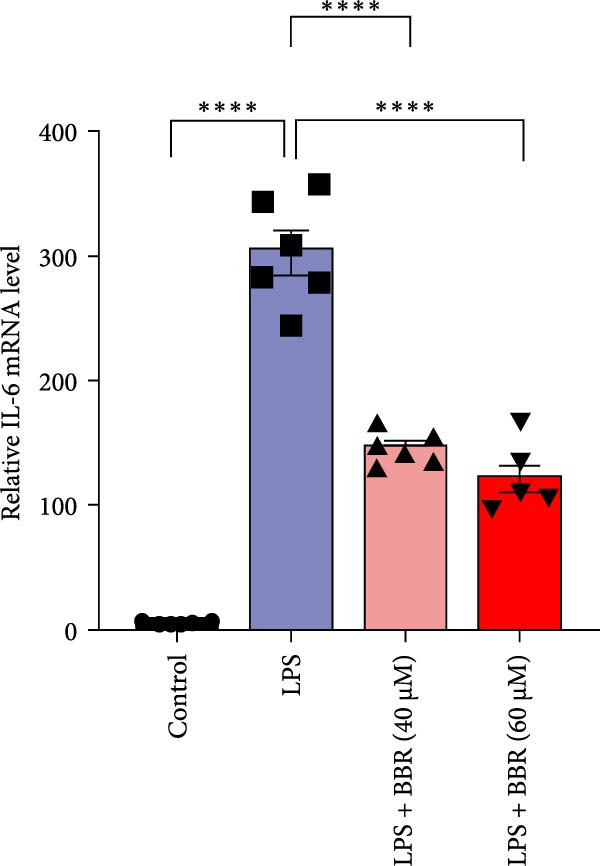
(E)

**Figure:**
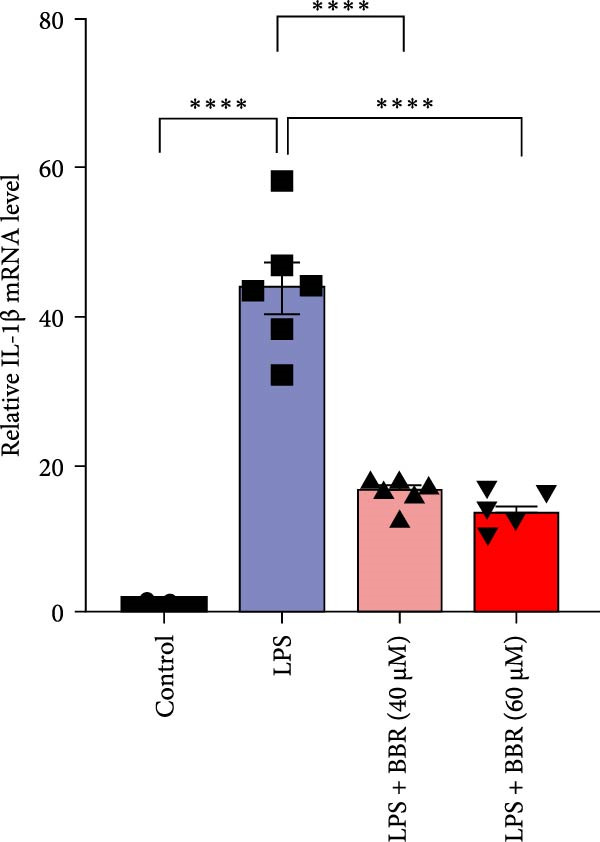
(F)

**Figure:**
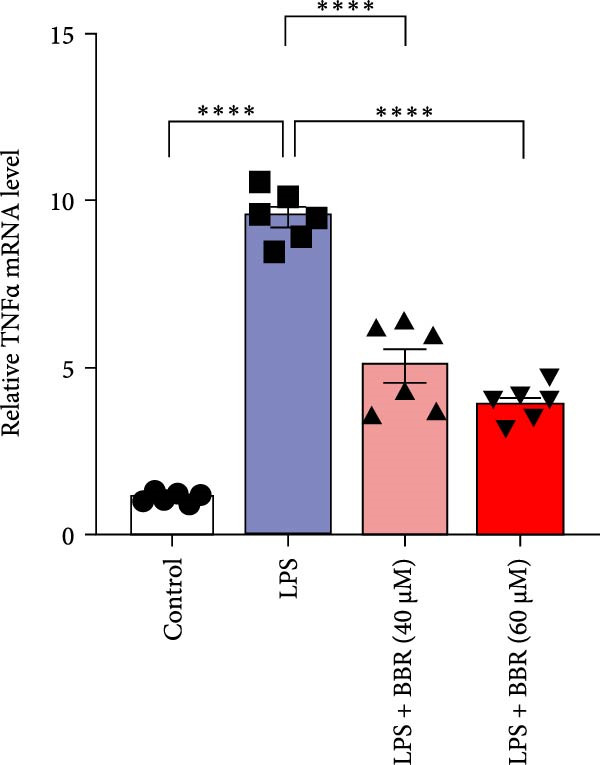
(G)

Figure 10BBR modulates the MAPK signaling pathways in LPS‐stimulated BV2 microglial cells. The cells were plated in 12‐well plates, incubated with BBR for 1 h, and then stimulated for 24 h with LPS (1 µg/mL). (A) Western blot analysis was performed to evaluate the protein levels of pJNK/JNK (B), pERK1/2/ERK1/2 (C), and pp38‐MAPK/p38‐MAPK (D) in LPS‐stimulated BV2 cells across different treatment groups. GAPDH was used as a loading control.  ^∗^ *p* < 0.05,  ^∗∗^ *p* < 0.01,  ^∗∗∗^ *p* < 0.001,  ^∗∗∗∗^ *p* < 0.0001.

**Figure:**
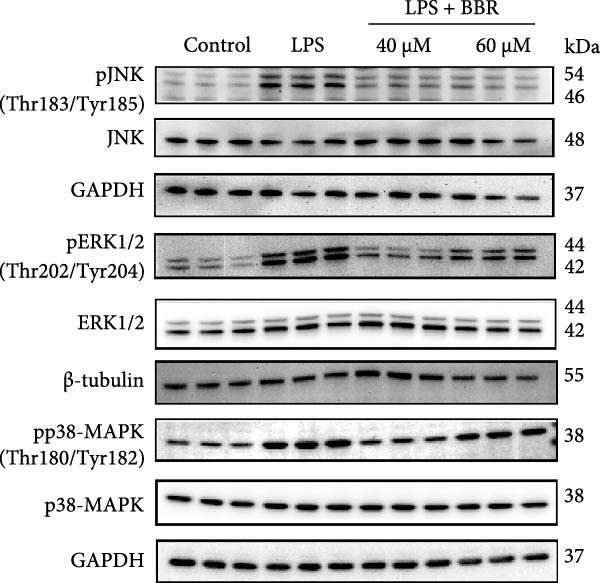
(A)

**Figure:**
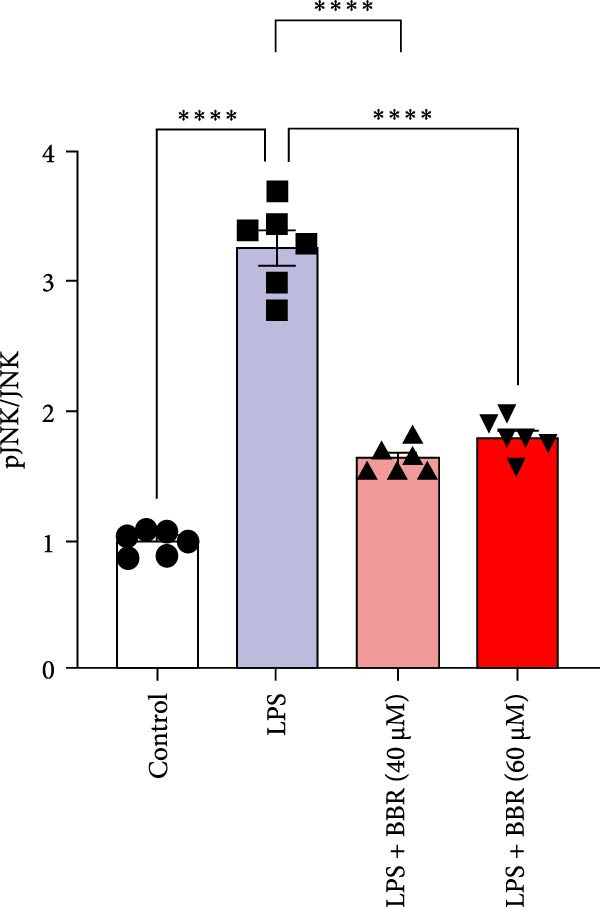
(B)

**Figure:**
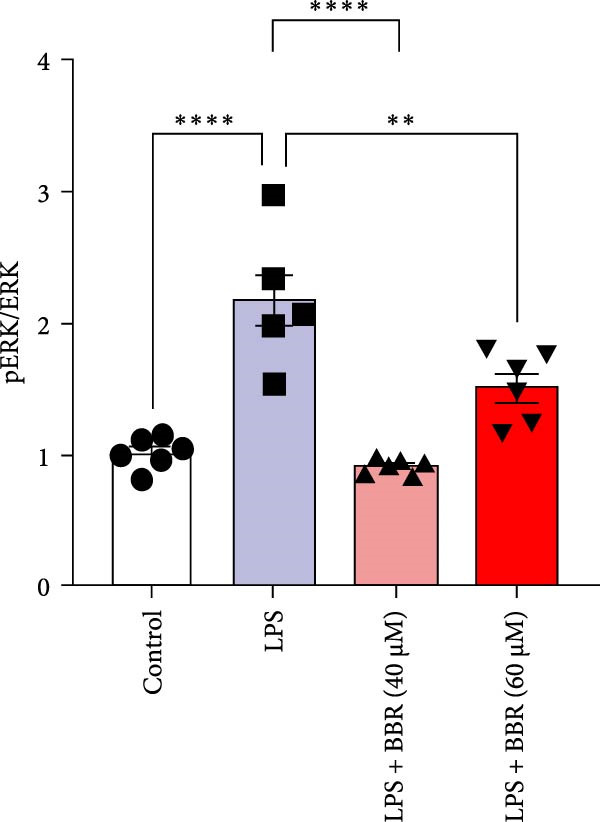
(C)

**Figure:**
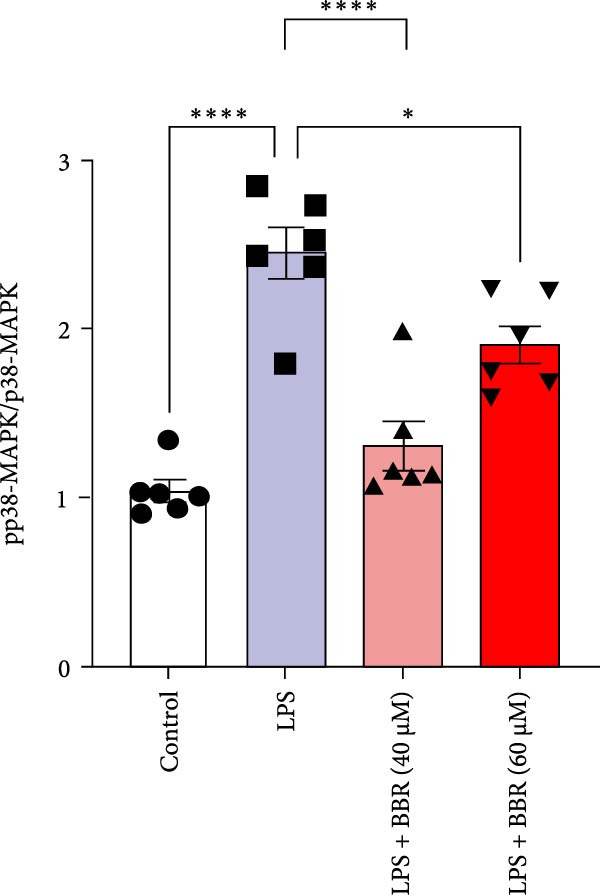
(D)

### 3.8. Molecular Docking of BBR With MAPK Target Proteins

This study also investigated the interaction between BBR and the key target protein of the MAPK signaling pathway. BBR indicated the highest degree of centrality within the network and interacted with critical target proteins in the MAPK signaling pathway, specifically P38MAPK, ERK2, JNK1, and JNK2 (Figure 11). Furthermore, BBR had good binding affinity with MAPK11 and ERK2 (Table 2), indicating that MAPK11 and ERK2 can serve as a potential primary target of BBR. These computational results demonstrate BBR’s preferential binding to multiple key components of the MAPK cascade, as visualized in the 3D/2D interaction models (Figure 11). Moreover, the MM‐GBSA calculations were performed to estimate the free binding energy between the ligand and the docking protein. The binding free energy of the MAPK family and BBR was obtained through the Glide docking module (Table 2). The results showed that both the MAPK family and BBR exhibited good binding free energy.

Figure 11The 3D and 2D molecular docking diagrams of the binding interactions between BBR and the MAPK family, specifically P38MAPK, ERK2, JNK1, and JNK2. (A) displays the molecular docking results for P38MAPK‐3GP0. (B) presents the molecular docking results for ERK2. (C) depicts the molecular docking results for JNK1. (D) shows the molecular docking results for JNK2. The purple structures represent the molecular configurations of the MAPK, ERK, JNK1, and JNK2 proteins, while the green molecule denotes BBR.

**Figure:**
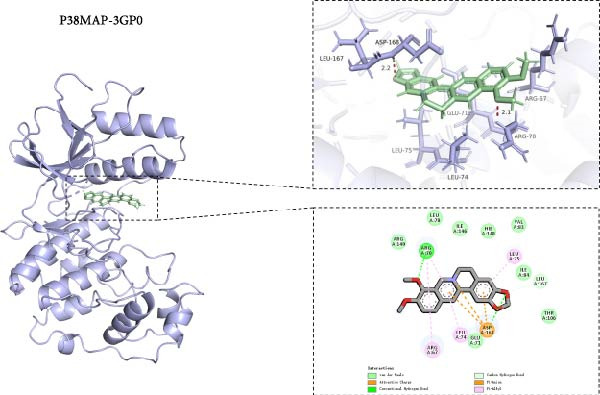
(A)

**Figure:**
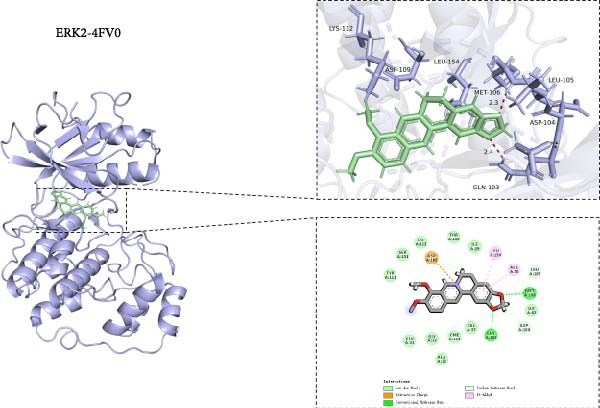
(B)

**Figure:**
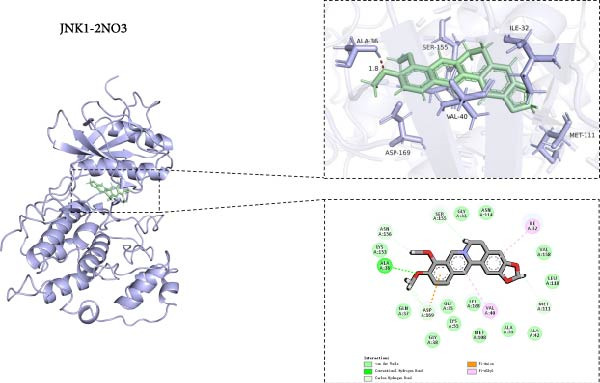
(C)

**Figure:**
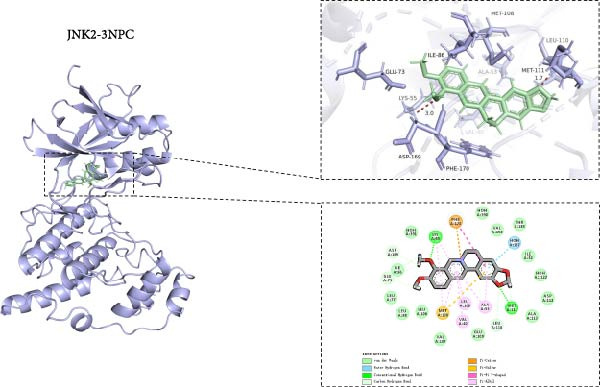
(D)

**Table 2:** The binding free energy of P38MAPK, ERK2, JNK1, JNK2, and BBR.

No.	Protein	PDB ID	Structure	Docking compound	Binding free energy (kcal/mol)
1	p38*β* (MAPK11)	3GP0	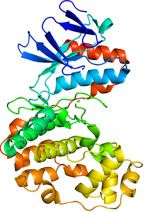	Berberine	−50.8694315478
2	ERK2	4FV0	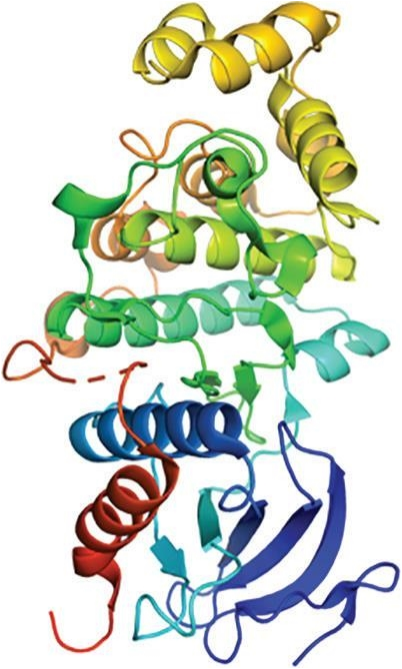	Berberine	−53.4311985359
3	JNK1	2NO3	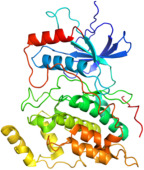	Berberine	−43.071775945
4	JNK2	3NPC	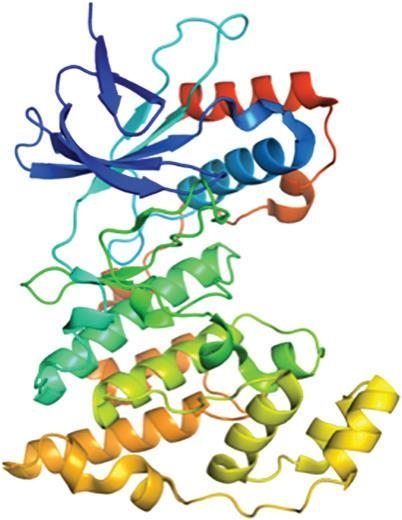	Berberine	−37.5772644833

*Note:* Description—evaluate the stability and binding affinity of the BBR, target protein complex based on the relative binding free energy calculated from MM‐GBSA.

## 4. Discussion

Several studies have indicated that cognitive deficits are associated with various pathological processes, such as peripheral and central neuroinflammation induced by surgical trauma, oxidative stress, hormonal homeostasis disruption (e.g., cortisol dysregulation), and anesthetic toxicity [40, 41]. Neuroinflammation is considered the main driver of cognitive deterioration, exerting its effect by directly mediating neuronal injury, synaptic loss, and neural network disruption, as well as via multiple signaling pathways associated with cognitive deficits [14, 42, 43]. Therefore, it is crucial to identify the primary mechanisms associated with neuroinflammation and identify effective pharmacological agents that target these mechanisms for developing novel strategies to treat cognitive deficits.

BBR, a quaternary ammonium alkaloid sourced from “*Coptis chinensis,”* has multiple pharmacological properties, including antibacterial, antioxidant, anti‐inflammatory, antiapoptotic, and neuroprotective activities [44, 45]. Recent studies have indicated the significance of BBR in inhibiting A *β*‐induced microglial activation by suppressing cytokine signaling [46]. Furthermore, BBR has been found to alleviate intestinal inflammation, reduce intestinal permeability, and improve gut microbiota composition in 5xFAD mice, removing A *β* plaque and improving spatial memory [47]. Moreover, BBR improves cognitive function in AD models by reducing tau hyperphosphorylation and enhancing tau autophagy [48]. Moreover, research suggests that BBR plays a significant role in the inflammatory response following peripheral nerve injury by modulating macrophage polarization [49]. However, despite these advances, the specific mechanisms underlying the anti‐neuroinflammatory effects of BBR against neuroinflammation‐induced cognitive impairment remain undetermined. Therefore, this study investigated how BBR modulates microglia‐mediated neuroinflammation in cognitive impairment.

The *in vivo* analyses indicated that only the high‐dose BBR treatment had significantly improved effects, attributable to the compound’s restricted water solubility, which led to inadequate oral and intraperitoneal absorption, limiting its therapeutic efficacy [33]. Treatment with high‐dose BBR restored dendritic spine density, preserved synaptic proteins (PSD95 and synaptophysin), and enhanced LTP in LPS‐challenged mice. These changes were associated with improved spatial memory in the MWM test. Furthermore, BBR shifted microglial polarization from the pro‐inflammatory M1 phenotype to the anti‐inflammatory M2 phenotype, as evidenced by a reduction in the number of Iba‐1 + /INOS + cells and an increase in Iba‐1^+^/Arg‐1^+^ populations. These data indicate that BBR mitigates neuroinflammation by inhibiting M1 microglial activation and facilitating M2 microglial polarization. It has been observed that imbalanced microglial phenotypic transformation promotes the progression of neurodegenerative diseases. Under pathological states, microglia might become hyperactivated, increasing the secretion of pro‐inflammatory cytokines and differentiation into the anti‐inflammatory M2 phenotype, which significantly contributes to cognitive impairment. In addition, the ability of microglia to co‐express both M1 and M2 phenotype markers indicates a transitional state and underscores their remarkable plasticity [50, 51]. Therefore, the dysregulation of microglial phenotypic polarization is the primary factor that promotes neuroinflammation and related brain injury [52–55].

This study also revealed that BBR improves cognitive impairment by suppressing the activation of key proteins within the MAPK signaling pathway, modulating microglial polarization. In addition, molecular docking confirmed a favorable binding energy between BBR and MAPK family proteins. Previous literature suggests that the MAPK signaling pathway, particularly the p38 and JNK pathways, is critical in mediating microglial activation and the subsequent release of inflammatory mediators [56, 57]. BBR has been observed to be significantly associated with MAPK‐mediated neuroinflammatory cascades; however, the complete upstream regulatory pathway remains elusive. Moreover, reactive oxygen species, the NF‐*κ*B pathway, and autophagy are crucial regulatory components that coordinately modulate MAPK activation [57, 58]. Future studies should investigate the complex association between these signaling pathways to identify new therapeutic targets and techniques for treating neuroinflammation‐related cognitive disorders. The findings of this study provide novel insights into the pharmacological mechanisms of BBR, highlighting its role in maintaining microglial homeostasis and mitigating inflammation‐associated cognitive impairment.

## 5. Conclusion

In summary, this study demonstrated that BBR significantly alleviates neuroinflammation, reduces neuronal apoptosis, and improves synaptic plasticity, ameliorating cognitive impairment‐associated pathologies.

## 6. Limitations

This study has several limitations. (1) The use of a single‐dose intrahippocampal LPS injection model may not adequately capture the complexities of human surgical trauma, necessitating validation through diverse modeling strategies. (2) The potential interactions between BBR‐mediated anti‐inflammatory properties and other pathways, such as the crosstalk between MAPK signaling and NF‐*κ*B, as well as autophagy, remain poorly understood. (3) This study primarily focused on the role of microglia in BBR‐mediated improvements in neuroplasticity and did not investigate the potential contributions of other glial cells, such as astrocytes and oligodendrocytes. These cells may collectively influence the complex microenvironment via which BBR exhibits its neuroprotective effects. Future research should focus on examining these factors to comprehensively elucidate the cellular mechanisms responsible for BBR‐induced improvement in neuroplasticity. Despite these limitations, the current study offers significant data validating the therapeutic efficacy of BBR in neuroinflammation‐related cognitive disorders.

## Ethics Statement

Ethical approval was obtained from the Institutional Animal Care and Use Committee at Southwest Medical University (Permit number: SYXK 2021‐0264).

## Disclosure

All authors confirmed the manuscript.

## Conflicts of Interest

The authors declare no conflicts of interest.

## Author Contributions

Yang Yu and Wei Dong conceived and designed the project. Lirong Jiang, Ruiyi Liao, Jiaxin Wang, Yuan Yang, Jiaojiao Sun, Li Liu, and Yiwei Wang performed all the experiments and prepared figures. Yang Yu participated in drafting and finalizing the manuscript. Lirong Jiang and Ruiyi Liao contribute equally to this work.

## Funding

This work were supported by the Sichuan Science and Technology Program (Grants 2025ZNSFSC1564 and 2023NSFSC0593), the Open Fund of Key Laboratory of Medical Electrophysiology (Grants KeyME‐2025‐07 and KeyME‐2024‐06), and the Technological Plan Program of Luzhou (Grant 2024JYJ011).

## Data Availability

The data that support the findings of this study are available from the corresponding author upon reasonable request. The authors confirm that the data supporting the findings of this study are available within the article.
